# Selective and competitive functions of the AAR and UPR pathways in stress-induced angiogenesis

**DOI:** 10.1038/s41421-021-00332-8

**Published:** 2021-10-26

**Authors:** Fan Zhang, Qi-Yu Zeng, Hao Xu, Ai-Ning Xu, Dian-Jia Liu, Ning-Zhe Li, Yi Chen, Yi Jin, Chun-Hui Xu, Chang-Zhou Feng, Yuan-Liang Zhang, Dan Liu, Na Liu, Yin-Yin Xie, Shan-He Yu, Hao Yuan, Kai Xue, Jing-Yi Shi, Ting Xi Liu, Peng-Fei Xu, Wei-Li Zhao, Yi Zhou, Lan Wang, Qiu-Hua Huang, Zhu Chen, Sai-Juan Chen, Xiao-Long Zhou, Xiao-Jian Sun

**Affiliations:** 1 Shanghai Institute of Hematology, State Key Laboratory of Medical Genomics, National Research Center for Translational Medicine (Shanghai), Ruijin Hospital Affiliated to Shanghai Jiao Tong University School of Medicine, Shanghai, China; 2 State Key Laboratory of Molecular Biology, CAS Center for Excellence in Molecular Cell Science, Shanghai Institute of Biochemistry and Cell Biology, Chinese Academy of Sciences, University of Chinese Academy of Sciences, Shanghai, China; 3 Department of Cardiology, Shanghai General Hospital, Shanghai Jiao Tong University School of Medicine, Shanghai, China; 4 School of Life Sciences & Biotechnology, Shanghai Jiao Tong University, Shanghai, China; 5 CAS Key Laboratory of Tissue Microenvironment and Tumor, Shanghai Institute of Nutrition and Health, Shanghai Institutes for Biological Sciences, University of Chinese Academy of Sciences, Chinese Academy of Sciences, Shanghai, China; 6 Key Laboratory of Systems Biomedicine, Ministry of Education, Shanghai Center for Systems Biomedicine, Shanghai Jiao Tong University, Shanghai, China; 7 Division of Human Reproduction and Developmental Genetics, Women’s Hospital, and Institute of Genetics and Department of Genetics, Zhejiang University School of Medicine, Hangzhou, Zhejiang China; 8 Stem Cell Program, Hematology/Oncology Program at Children’s Hospital Boston and Dana Farber Cancer Institute, Harvard Medical School, Boston, MA USA

**Keywords:** Stress signalling, tRNAs, Bioinformatics

## Abstract

The amino acid response (AAR) and unfolded protein response (UPR) pathways converge on eIF2α phosphorylation, which is catalyzed by Gcn2 and Perk, respectively, under different stresses. This close interconnection makes it difficult to specify different functions of AAR and UPR. Here, we generated a zebrafish model in which loss of threonyl-tRNA synthetase (Tars) induces angiogenesis dependent on Tars aminoacylation activity. Comparative transcriptome analysis of the *tars*-mutant and wild-type embryos with/without Gcn2- or Perk-inhibition reveals that only Gcn2-mediated AAR is activated in the *tars*-mutants, whereas Perk functions predominantly in normal development. Mechanistic analysis shows that, while a considerable amount of eIF2α is normally phosphorylated by Perk, the loss of Tars causes an accumulation of uncharged tRNA^Thr^, which in turn activates Gcn2, leading to phosphorylation of an extra amount of eIF2α. The partial switchover of kinases for eIF2α largely overwhelms the functions of Perk in normal development. Interestingly, although inhibition of Gcn2 and Perk in this stress condition both can reduce the eIF2α phosphorylation levels, their functional consequences in the regulation of target genes and in the rescue of the angiogenic phenotypes are dramatically different. Indeed, genetic and pharmacological manipulations of these pathways validate that the Gcn2-mediated AAR, but not the Perk-mediated UPR, is required for *tars*-deficiency induced angiogenesis. Thus, the interconnected AAR and UPR pathways differentially regulate angiogenesis through selective functions and mutual competitions, reflecting the specificity and efficiency of multiple stress response pathways that evolve integrally to enable an organism to sense/respond precisely to various types of stresses.

## Introduction

Stress-induced angiogenesis contributes enormously to both normal development and pathogenesis of various diseases including cancer, metabolic, and cardiovascular disorders^1^. Among many stress response pathways implicated in the regulation of angiogenesis, the amino acid response (AAR)^2^ and unfolded protein response (UPR) pathways^3,4^ are closely interconnected, as they converge on the common target, eukaryotic initiation factor 2 subunit α (eIF2α), the key regulator of protein translation^5,6^. Two kinases, general control nonderepressible 2 (Gcn2; also known as Eif2ak4)^7–9^ and protein kinase R-like endoplasmic reticulum (ER) kinase (Perk; also known as Eif2ak3)^10,11^, are responsible for transducing signals from AAR and UPR, respectively, to cause eIF2α phosphorylation. Furthermore, eIF2α can also be phosphorylated by a heme-regulated translational inhibitor (Hri; also known as Eif2ak1)^12,13^ and protein kinase RNA-activated (Pkr; also known as Eif2ak2)^14,15^, which respectively respond to the heme deprivation and viral infection stresses, adding dimensions of complexity to these regulatory mechanisms. All these stress response pathways have been referred to as the integrated stress response^16,17^. While it is possible that the cells/organisms can respond differentially to the diverse stresses, for example, by distinguishing which phosphorylated eIF2α is catalyzed by which kinase, clear evidence for this specificity remains to be seen. Thus, even though numerous insightful studies have been conducted, such a close interconnection between AAR and UPR makes it difficult to clearly distinguish their different contributions in angiogenesis.

As a major translation regulatory mechanism, the stress-induced eIF2α phosphorylation leads to reduced global translation initiation coincident with the preferential translation of the transcription factor activating transcription factor 4 (ATF4)^5,6^. The phosphorylation of eIF2α prevents regeneration of the active form of the eIF2 complex, which controls translation initiation, and thereby inhibits global translation on one hand. On the other hand, the limited availability of functional eIF2 complex facilitates the selective translation of ATF4 through a ribosome re-initiation mechanism coordinated by two upstream open reading frames (uORFs) of the *ATF4* mRNA^18^. The upregulated ATF4 plays important role in both AAR and UPR by transcriptional regulation of a large number of target genes that are associated with various physiological and pathological functions including angiogenesis^19,20^.

The AAR and UPR pathways are evolved to sense and respond to two different types of stresses, namely amino acid starvation as nutritional stress and unfolded proteins as a cellular endogenous stress, respectively^21,22^. Therefore, although AAR and UPR share the common targets eIF2α and the downstream translation regulatory machinery, they must also exert unique functions possibly through both overlapping and divergent mechanisms, which could be reflected by their differentially regulated target genes^22^. Indeed, a previous study using an in situ perfused mouse liver model has identified some differentially regulated genes by Gcn2 and Perk^23^, thus implying the possibility of distinguishing AAR and UPR initially by comparing gene expression profiles. Nevertheless, a systematic comparative study of AAR and UPR in a biological process such as angiogenesis is lacking.

In this study, we generated a zebrafish angiogenic model harboring a loss-of-function mutation of the *threonyl-tRNA synthetase* (*tars*) gene. Tars belongs to a family of evolutionarily conserved enzymes, aminoacyl-tRNA synthetases (aaRSs), which control the first step of protein translation through coupling specific amino acids with their cognate tRNAs^24,25^. Notably, several aaRSs have been implicated in the regulation of angiogenesis, and some relevant phenotypes have been attributed to activation of UPR^4,26^ or explained by noncanonical functions that are probably associated with the splice variants of some aaRSs^27,28^. However, the precise mechanism remains elusive, and it is unclear whether AAR, which can be activated by uncharged tRNAs^29,30^ and stalled ribosomes^31,32^, is also activated and, if so, whether both AAR and UPR are required and to what extent each of them contributes to this process. To compare the role of AAR and UPR in *tars*-deficiency-induced angiogenesis, we designed an approach combining comparative gene expression profiling and quantitative phenotype analysis of zebrafish embryos with different genotypes and under specific treatments. These studies allowed us to identify the different roles of AAR and UPR in stress-induced angiogenesis.

## Results

### Angiogenic phenotypes of the *tars* mutants and requirement of Tars aminoacylation activity

**Fig. 1:**
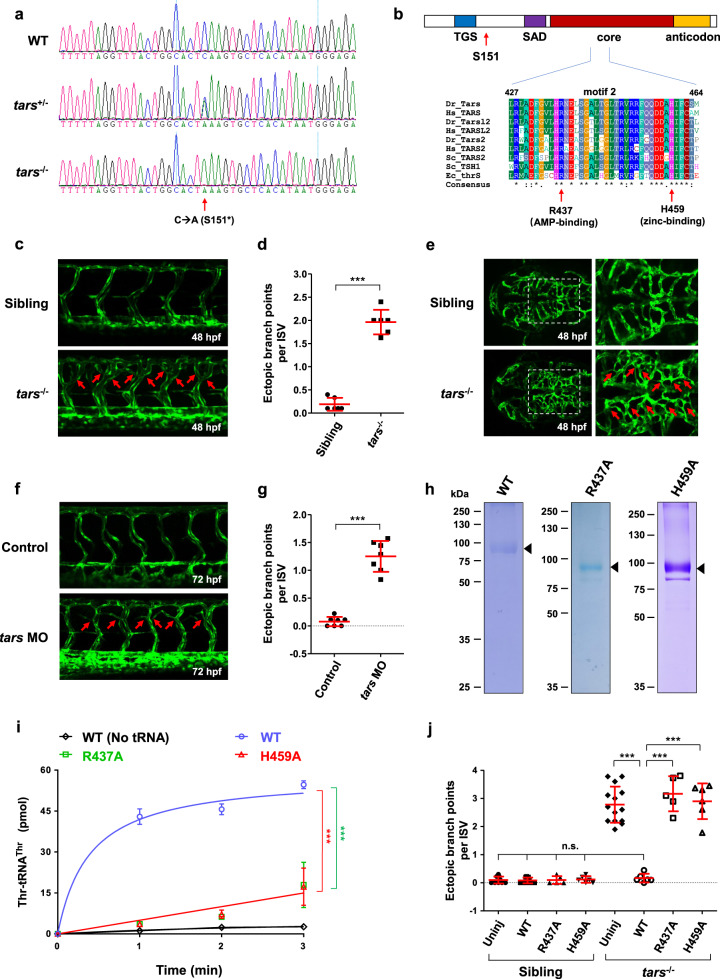
Angiogenic phenotypes of the *tars* mutants and the requirement of Tars aminoacylation activity. **a** Sequencing results of the wild-type (WT) and the *tars* mutated heterozygous (*tars*^+/−^) and homozygous (*tars*^−/−^) zebrafish embryos. The arrow denotes the C-to-A nonsense mutation that generates a premature stop codon after serine 151 (S151*). **b** Domain architecture of the Tars protein, showing the position of serine 151 (S151; arrow), where the mutation-introduced stop codon predicts a deletion of the downstream SAD, core, and anticodon domains. Also shown is the alignment of the amino acid sequences of a signature motif (motif 2) within the core domain. Two evolutionarily conserved residues, R437 and H459, which are required for the aminoacylation activity and are directly involved in AMP and zinc binding, respectively, were chosen for functional studies. The alignment includes Tars and its homologs (i.e., Tars2 and Tarsl2, etc.) from humans (*Hs*, *Homo sapiens*), zebrafish (*Dr, Danio rerio*), yeast (*Sc*, *Saccharomyces cerevisiae*), and enterobacterial (*Ec*, *Escherichia coli*). **c** Confocal microscopy images of EGFP-labeled blood vessels in the trunk of the WT and *tars*^−/^^−^ zebrafish embryos at 48 hpf. **d** Quantification and statistical analysis of the ectopic branch points per ISV of the *tars*^−/^^−^ and sibling embryos. **e** An abnormal increase of branches in the hindbrain capillaries of the *tars*^−/^^−^ embryos compared with the siblings. Magnified views of the dashed boxed regions are shown on the right. **f** Increased branch points in the ISVs caused by *tars* MO in WT embryos. In panels **c**, **e**, **f**, the arrows denote ectopic branch points of the vessels. **g** Quantification and statistical analysis of the ectopic branch points per ISV of the control and Tars knockdown embryos. **h** Coomassie blue staining of WT and mutant zebrafish Tars proteins, which were purified with His-tag from *E. coli*. **i** Aminoacylation activity assays with the purified proteins, showing the nearly abolished enzymatic activities of the R437A and H459A mutants compared with the WT Tars protein. **j** Rescue of the *tars*^−/^^−^ angiogenic phenotype by injection of the WT, but not the inactivation mutant, *tars* mRNAs. Note that, for the *tars*^−/^^−^ embryos, injection of the WT *tars* mRNA, but not the R437A or H459A mutant mRNA, significantly reduced the ectopic branch points per ISV compared with the uninjected *tars*^−/^^−^ embryos (uninj). In contrast, for the WT embryos, injection of these WT, R437A, and H459A mRNAs showed no effect on the ISVs compared to the uninjected controls. Also, note that the phenotypic rescue by the WT *tars* mRNA was almost complete because quantification of the branch points of the injected embryos showed no difference compared with the WT embryos. In panels **d**, **g**, **i**, **j**, data are presented as means ± SD; two-tailed *t-*test; ***P < 0.001; n.s., not significant.

A zebrafish recessive mutant line with angiogenic phenotypes was generated in our *N*-ethyl-*N*-nitrosourea (ENU) screening project as described previously^33,34^. Whole-exosome sequencing of the family of this line identified that its *tars* gene carries a C-to-A mutation that produces a stop codon (TAA) after the residue serine 151 (S151*; Fig. 1a). Ectopic expression of the *tars* mutant cDNA fused with a Flag-tag at the N-terminus showed that it only encoded a truncated protein (Supplementary Fig. S1). This Tars mutant protein should be nonfunctional because its major domains responsible for tRNA-binding (second additional domain; SAD), catalytic activity (core domain), and anticodon recognition (anticodon domain) are all deleted (Fig. 1b). This *tars* mutant line was crossed with the Tg(*flk1:EGFP*) transgenic line^35^ to visualize blood vessels in vivo. Although the *tars* S151* homozygous mutation made *tars*^−/−^ zebrafish lethal at around 6 days-post-fertilization (dpf), the angiogenic phenotypes of the *tars*^−/−^ embryos were evident after 48 hours-post-fertilization (hpf), as their intersomitic vessels (ISVs) exhibited frequent ectopic branching, especially in the dorsal portion (Fig. 1c), suggesting a defect in vascular patterning^36^. Quantification of the frequency of the ectopic branch points per ISV^37^ showed a statistically significant difference between the *tars*^−/−^ and the sibling embryos (Fig. 1d). Abnormal branches were also clearly observed in the hindbrain capillaries (Fig. 1e), suggesting that the whole body rather than specific organs/tissues contained the angiogenic defect. These angiogenic phenotypes were faithfully recapitulated by a morpholino-mediated knockdown of Tars (*tars* MO) in the wild-type (WT) embryos (Fig. 1f, g).

To determine whether the aminoacylation activity of Tars is critical for this function, we generated alanine-mutations in either of two evolutionarily conserved residues in the aminoacylation activity site, namely arginine 437 (R437) and histidine 459 (H459) (Fig. 1b), which mediate the adenosine monophosphate (AMP) and zinc binding, respectively^38^. In vitro aminoacylation activity assays with the purified WT and mutant Tars proteins (Fig. 1h) showed that the R437A and H459A mutations nearly abolished the aminoacylation activity of Tars (Fig. 1i). Phenotypic rescue experiments by injection of the WT or mutant *tars* mRNAs into the *tars*^−/^^−^ embryos showed that, while the mutant angiogenic phenotypes were completely suppressed by the WT Tars, the R437A and H459A mutants could not rescue these phenotypes (Fig. 1j). Thus, these results indicate that the function of Tars in regulating angiogenesis is dependent on its aminoacylation activity.

### Comparative transcriptome profiles reveal that the Gcn2-mediated AAR, but not the Perk-mediated UPR, is activated in the *tars* mutant embryos

**Fig. 2:**
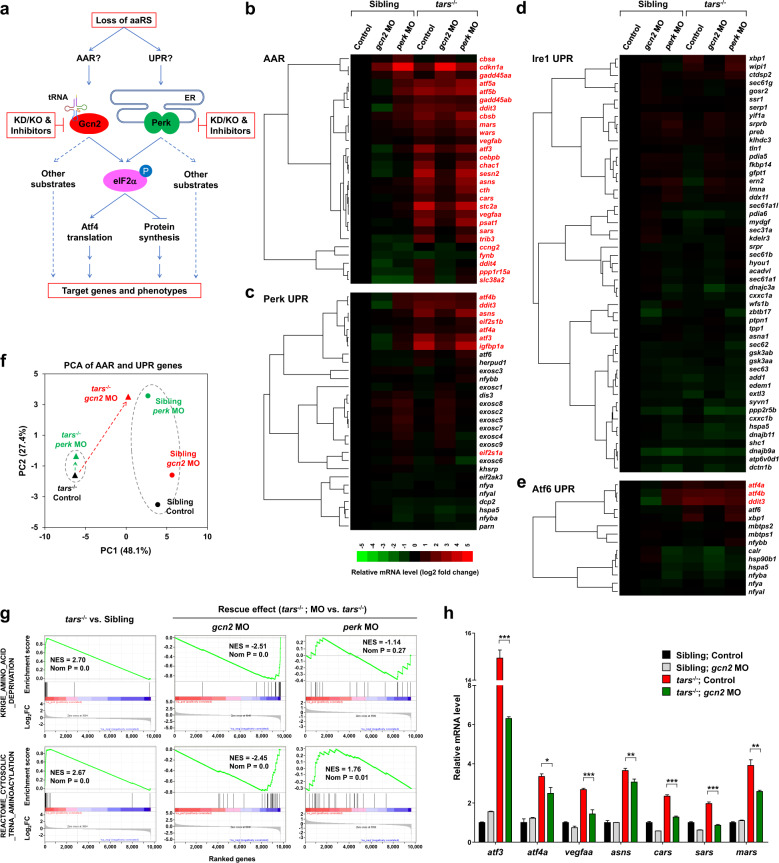
Differential and combinatorial regulation of the AAR- and UPR-associated genes by functional loss of Tars, Gcn2, or Perk. **a** Schematic overview of study design. Both AAR and UPR may regulate the angiogenic phenotypes induced by deficiency of aminoacyl-tRNA synthetases (aaRSs). To distinguish between them, the proposed experimental strategies (in red boxes) were based on the *tars* mutant and the siblings; knockdown (KD), knockout (KO), and pharmacological inhibition of Gcn2 and Perk were used, and systematic gene expression profiling of the zebrafish embryos with various genotypes and treatments was performed. **b**–**e** Hierarchical clusters and heatmaps of the expression levels of the genes in the AAR gene set (**b**) and in the Perk-, Ire1-, and Atf6-mediated UPR gene sets (**c**–**e**). The data were produced by RNA-seq analyses of 36 hpf homozygous *tars*-mutated (*tars*^−/^^−^) embryos and their siblings from the same litters (sibling) that were treated with indicated morpholino (MO). The gene symbols written in red are known to be involved in AAR, even though some of them are also listed in the UPR gene sets (for further information and references, see Supplementary Tables S1–S4). The color bar indicates relative expression levels. Note that the upregulated genes in *tars*^−/^^−^ mostly fall into the AAR category (written in red), whereas the UPR genes are largely unchanged, except for those shared in both pathways. **f** PCA of the AAR- and UPR-associated genes of the *tars*^−/^^−^ (triangles) and siblings (circles) treated with *gcn2* MO (red) or *perk* MO (green), compared with the control groups (black), respectively. Note that *gcn2* MO, but not *perk* MO, significantly reversed the major component (PC1) score of *tars*^−/^^−^, whereas the *perk* MO only altered the PC2 score of the siblings but has a very subtle effect on the *tars*-mutants. **g** GSEA results showing strong enrichments of the AAR and tRNA aminoacylation genes in the *tars*^−/^^−^ embryos, which can be reversed by *gcn2* MO but not *perk* MO. **h** RT-qPCR analysis of the representative genes that are activated in the *tars*^−/^^−^ embryos and are downregulated by *gcn2* MO. Data are presented as means ± SD of triplicate reactions. ***P < 0.001; **P < 0.01; *P < 0.05. The *P* values for the increased expression of all these genes in *tars*^−/^^−^ relative to the siblings are less than 0.001 (not shown).

The *tars* mutant zebrafish provides an ideal in vivo model for dissecting the regulatory mechanism of the stress-induced angiogenesis because of its advantages including (i) direct visualization and quantification of the angiogenic phenotypes, (ii) easy application of genetic and pharmacological manipulations of the AAR and UPR pathways, and (iii) plenty of material for gene expression and proteomic analysis. We therefore designed an approach to compare the AAR and UPR pathways in the same experimental system by combining comparative transcriptome profiling and quantitative phenotype analysis of the zebrafish embryos in both normal and stress conditions (Fig. 2a). To this end, we first performed RNA-seq analyses of the *tars*^−/−^ and sibling embryos, as well as those with knockdown of either Gcn2 or Perk by morpholinos in both genotypes. Notably, the functional effects of these morpholinos were validated based on similar phenotypes and molecular features between the morpholino-injected embryos and the genetic knockout lines (see below). All the morpholinos used in this study were also either verified by the GFP-fusion reporter assay^39^ (Supplementary Fig. S2) or reported in previously published studies^4,34,40^. We found that the AAR-associated genes were dramatically activated in the *tars*^−/^^−^ embryos (Fig. 2b; Supplementary Table S1). In contrast, the majority of genes associated with the three UPR sub-pathways (i.e., the Perk-, Ire1-, and Atf6-mediated UPR pathways)^3^ remained inactive, except for very few genes (e.g., *atf3*, *atf4a*/*b*, *ddit3*, *asns*, *eif2s1*, and *igfbp1*) that are known to be shared between AAR and UPR^41–43^ (Fig. 2c–e; Supplementary Tables S2–S4). These results suggest that AAR, but not UPR, is activated in the *tars*^−/^^−^ embryos. In support of this notion, knockdown of the AAR-associated kinase Gcn2 in *tars*^−/^^−^ largely repressed the activated genes, whereas the Perk knockdown showed very little effect (Fig. 2b–e). Principal component analysis (PCA) of gene expression results also showed that knockdown of Gcn2, but not Perk, almost reversed the PC1 score of *tars*^−/^^−^ back to that of the siblings (Fig. 2f), reflecting a rescue of a large portion of gene expression variances. Furthermore, overall gene set enrichment analysis (GSEA) of the RNA-seq data and RT-qPCR validations of representative genes indicated that the upregulated genes in *tars*^−/^^−^ were extremely enriched in the genes associated with AAR and tRNA aminoacylation and that this enrichment was largely reversed by knockdown of Gcn2, but not Perk (Fig. 2g, h). The observation that several of the upregulated genes in *tars*^−/^^−^ were reversed incompletely to the basal level by Gcn2 knockdown (Fig. 2b, c, e, h) may reflect partial induction of other eIF2α phosphorylation-independent (e.g., p53) pathways, which may also regulate some of these genes^44–46^. In support of this possibility, our results showed that some p53 target genes such as *cdkn1a* and *gadd45aa/b* were upregulated in the Gcn2 knockdown *tars*^−/^^−^, as well as the Perk knockdown normal embryos (Fig. 2b).

### Perk functions predominantly in normal development but its function might be overwhelmed by the stress-induced activation of Gcn2

In contrast to the apparently dispensable role of Perk in *tars*^−/^^−^, knockdown of Perk in normal (i.e., the sibling) embryos led to a significant gene expression alteration. As shown in the PCA results, knockdown of Perk in the siblings, but not in the *tars*^−/^^−^ embryos, caused a dramatic shift of the PC2 score (Fig. 2f, indicated by dotted ovals), suggesting that PC2 accounts for expression variance of the genes associated with Perk function in normal development. We further compared the gene expression profiles of different types of embryos and paid attention to the genes associated with RNA degradation because the Perk-mediated UPR had been specifically implicated in the regulation of nonsense-mediated mRNA decay^47^. Indeed, our GSEA results showed that: (i) the KEGG_RNA_DEGRADATION gene set was significantly enriched in the upregulated genes by Perk knockdown in normal embryos (Supplementary Fig. S3a, left), whereas Gcn2 knockdown (Supplementary Fig. S3a, middle) or *tars* knockout (Supplementary Fig. S3a, right) showed no significant enrichment; and (ii) when the Perk knockdown was performed in *tars*^−/^^−^, the enrichment of this gene set was completely abolished (Supplementary Fig. S3b). These results suggest that Perk mainly functions in homeostatic states (i.e., normal embryos) but, in the stress condition (i.e., *tars*^−/^^−^ embryos) its function might be largely overwhelmed by activation of the Gcn2-mediated AAR.

### Selective activation of Gcn2 or Perk leading to functionally distinct eIF2α phosphorylation

**Fig. 3:**
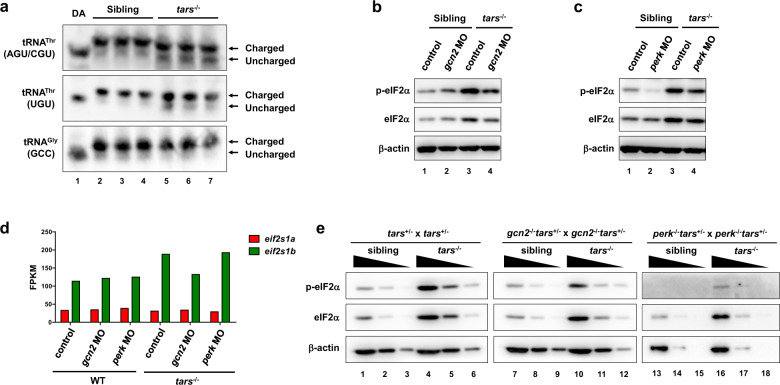
Selective activation of Gcn2 and Perk in stress condition and normal development. **a** Northern blot results showing the increased uncharged tRNA^Thr^ in the *tars*^−/^^−^ embryos compared with the siblings. Charged and uncharged tRNAs (upper and lower bands, respectively) were separated in an acid polyacrylamide/urea gel system; the three types of tRNA^Thr^ were hybridized with specific probes that could recognize tRNA^Thr^(AGU/CGU) or tRNA^Thr^(UGU). The tRNA^Gly^(GCC) was used as a negative control. Deacylated tRNAs (DA) were used to mark the migration position of the uncharged tRNAs on the gels. **b**, **c** Immunoblot analysis of the phosphorylated eIF2α (p-eIF2α) and total eIF2α upon morpholino-mediated knockdown of Gcn2 or Perk in the WT and *tars*^−/^^−^ embryos. β-actin was used as the loading control. Note that *gcn2* MO and *perk* MO reduced the phosphorylated eIF2α in the *tars*^−/−^ embryos to a comparable extent, whereas only *perk* MO reduced the eIF2α phosphorylation in the WT embryos. **d** Expression levels of the mRNAs of the two zebrafish eIF2α-coding genes, *eif2s1a* and *eif2s1b*, in the embryos of indicated genotypes. Presented are fragments per kilobase per million mapped reads (FPKM) values from RNA-seq data. Note that *eif2s1b* is upregulated in the *tars*^−/^^−^ embryos, which explains the accordingly increased protein levels as indicated by the immunoblot results. **e** Immunoblot analysis of the phosphorylated and total eIF2α level in the gene knockout embryos, which were produced by crossing the indicated mutant lines. Each sample was loaded in a 3-fold serial dilution to facilitate quantification. Note that the basal level of p-eIF2α was hardly detectable in the *perk*^−/^^−^ “normal” (sibling) embryos (lanes 13–15), and that the p-eIF2α in the *gcn2*^−/^^−^*tars*^−/^^−^ embryos was decreased, but not eliminated, compared with the *tars*^−/^^−^ embryos (compare lanes 7–12 with 1–6).

We then set out to clarify the mechanisms underlying the activation of Gcn2 in the *tars*^−/^^−^ embryos and the different gene-regulating functions of Gcn2 and Perk in different conditions. Although the stimuli for Gcn2 activation in higher eukaryotes has still been under debate^48^, previous yeast studies provide strong evidence that uncharged tRNAs can directly bind to, and activate, Gcn2^29,30^. We reasoned that the *tars* deficiency could cause accumulation of uncharged tRNA^Thr^ due to insufficient tRNA^Thr^ threonylation. To test this possibility, we isolated the total tRNAs from the *tars*^−/^^−^ and sibling embryos and subjected them to an acid polyacrylamide/urea gel system to separate the aminoacylated (charged) and uncharged tRNAs, followed by Northern blot to distinguish the different tRNAs. As a result, a significant increase of uncharged tRNA^Thr^ (including the tRNA^Thr^ (AGU/CGU) and tRNA^Thr^ (UGU) isoacceptors) was detected in the *tars*^−/^^−^ embryos compared with the siblings (Fig. 3a). In contrast, as a negative control, the aminoacylation ratio for tRNA^Gly^(GCC) was not changed (Fig. 3a). These results indicated that the loss of Tars specifically reduced the aminoacylation of tRNA^Thr^, but not other tRNA isoacceptors. However, it was clear that this threonylation was not completely blocked, probably because homologous proteins of Tars (e.g., Tars2 and Tarsl2) still exist and they may compensate for this reaction to a certain extent.

Consistent with the accumulation of uncharged tRNA^Thr^ in *tars*^−/^^−^ embryos, as well as the above-described gene expression patterns, our immunoblot analysis indeed showed that the eIF2α phosphorylation in *tars*^−/^^−^ embryos were significantly increased and that this increase was substantially suppressed by knockdown of Gcn2 (Fig. 3b). Notably, the total protein level of eIF2α was also increased upon Gcn2 activation (Fig. 3b), which in fact was caused by an upregulation of the eIF2α mRNA (see below for further analysis). Meanwhile, this analysis also showed that the normal embryos had a considerable “basal” level of eIF2α phosphorylation, which was not decreased by Gcn2 knockdown (Fig. 3b), suggesting that the basal eIF2α phosphorylation was catalyzed by other kinase(s). Indeed, knockdown of Perk, but not Gcn2, in the normal embryos eliminated this basal eIF2α phosphorylation (Fig. 3c), thus further supporting the idea that Perk functions predominantly in normal development.

As mentioned above, besides these regulations at the protein phosphorylation level, we observed an upregulation of the mRNA of *eif2s1b* (the major eIF2α-coding gene of zebrafish) (Fig. 3d), which explained the increase of total eIF2α protein in the *tars*^−/^^−^ embryos (Fig. 3b–d). As the upregulation of both mRNA and protein of eIF2α could be largely suppressed by knockdown of Gcn2, but not Perk (Fig. 3b, d), it was very likely that *eif2s1b* was one of the target genes of Gcn2-mediated AAR and, therefore, that eIF2α upregulation served as a secondary event upon Gcn2 activation. To examine whether the increase of total eIF2α per se was sufficient to cause the angiogenic phenotypes, we overexpressed eIF2α by injecting *eif2s1a* or *eif2s1b* mRNAs into the embryos (Supplementary Fig. 4a). The results showed that this eIF2α overexpression had no effect on the overall eIF2α phosphorylation or the angiogenic development (Supplementary Fig. 4b, c). On the other hand, knockdown of eIF2α by morpholinos of *eif2s1a* and *eif2s1b* in the *tars*^−/^^−^ embryos very slightly, albeit not significantly, affected the angiogenesis (Supplementary Fig. 4d–f). Thus, these results suggest that the angiogenic phenotypes are not exactly dependent on the total eIF2α protein level or its overall phosphorylation, but on which kinase(s) were catalyzing the phosphorylation of the proper eIF2α molecules.

Of note, these changes of eIF2α phosphorylation could be well-validated in gene knockout zebrafish lines. For example, immunoblot analysis of the *perk*^−/^^−^ and *gcn2*^−/^^−^ embryos, as well as those produced by breeding them with the *tars* mutants, showed that the eIF2α phosphorylation level was dramatically decreased in the *perk*^−/^^−^ embryos with WT *tars* (Fig. 3e; Supplementary Fig. S5), while *gcn2* knockout reduced, but not eliminated, the eIF2α phosphorylation in the *tars*^−/^^−^ embryos (Fig. 3e). Interestingly, these results also indicated that in the *tars*^−/^^−^ embryos, Gcn2 and Perk knockdown/knockout both reduced eIF2α phosphorylation to a comparable extent (Fig. 3b, c; and see Supplementary Fig. S6 for serial dilution immunoblot analysis); however, they showed dramatically different phenotypes and gene alterations, thus suggesting that the phosphorylated eIF2α molecules catalyzed by the different kinases should be functionally distinct.

### Functional requirement of AAR, but not UPR, for the *tars*-deficiency-induced angiogenesis

**Fig. 4:**
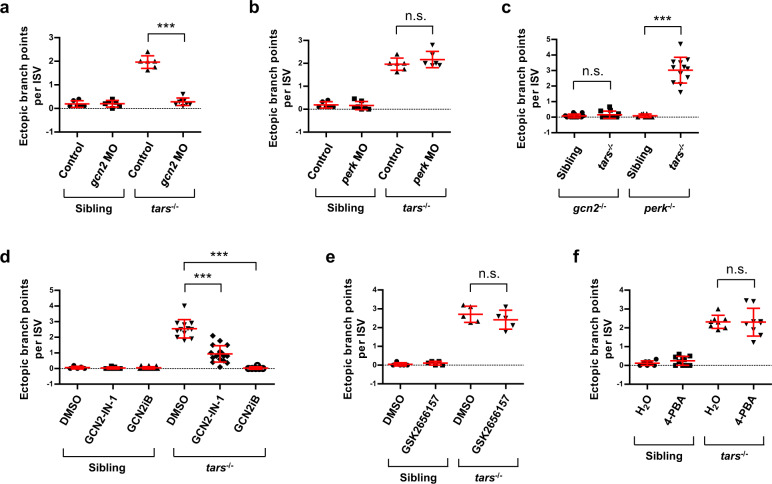
Genetic and pharmacological inhibition of AAR, but not UPR, suppresses the *tars*-deficiency-induced angiogenesis. **a**, **b** Quantification and statistical analysis of the ectopic branch points per ISV of the *tars*^−/^^−^ and sibling embryos that were treated with *gcn2* MO or *perk* MO. **c** Knockout of *gcn2*, but not *perk*, suppressed the *tars*-deficiency-induced angiogenesis. The embryos were produced by self-cross of *gcn2*^−/^^−^*tars*^+/^^−^ or *perk*^−/^^−^*tars*^+/−^ lines, and the *tars*^−/^^−^ ones were compared with their siblings in the same litter. **d** Pharmacological inhibition of Gcn2 by GCN2-IN-1 and GCN2iB significantly suppressed the angiogenic phenotypes of the *tars*^−/^^−^ embryos. **e** Inhibition of Perk by Perk-specific inhibitor GSK2656157 showed no significant rescue effect on the angiogenetic phenotypes. **f** Attenuation of UPR by 4-phenylbutyrate (4-PBA) could not rescue the angiogenetic phenotypes. In panels **d**–**f**, the solvents DMSO and H_2_O were applied as negative controls, respectively. In the statistical analyses, data are presented as means ± SD; two-tailed *t*-test; ***P < 0.001; n.s., not significant.

Given that the gene expression analysis indicated the activation of AAR, but not UPR, in the *tars*^−/^^−^ embryos, we next investigated whether the activated AAR was functionally required for the mutant angiogenic phenotypes and whether UPR, albeit at a basic level, could also contribute to the phenotypes. To address these questions, we first performed morpholino-mediated knockdown of Gcn2 or Perk in the *tars*^−/^^−^ and sibling embryos and quantitively analyzed their blood vessels that were labeled with the *flk1* promoter-driven EGFP. The results showed that knockdown of Gcn2, but not Perk, in the *tars*^−/−^ embryos reduced the vessel branch points to a level similar to that in the siblings (Fig. 4a, b), indicating rescue of the angiogenesis from the mutant phenotypes. Meanwhile, crossing the *tars* mutants with *gcn2*^−/^^−^ or *perk*^−/^^−^ lines showed that, only in the absence of *gcn2*, the *tars* deficiency failed to induce abnormal angiogenesis (Fig. 4c).

Consistent with this genetic analysis, pharmacological inhibition of Gcn2 by specific inhibitors GCN2-IN-1^49^ or GCN2iB^50^ significantly rescued the angiogenesis as well (Fig. 4d), whereas inhibition of Perk by GSK2656157^51^ showed no rescue (Fig. 4e). Furthermore, as 4-phenylbutyrate (4-PBA) had been shown to act as a chemical chaperone to suppress UPR in various conditions^52,53^, we used 4-PBA to attenuate remaining UPR activities, if any, in the *tars*^−/−^ and sibling embryos, and the results showed that the 4-BPA treatment could neither rescue the angiogenesis in the *tars*^−/^^−^ nor cause abnormalities in the blood vessels of the sibling embryos (Fig. 4f). Thus, these results indicate that AAR, but not UPR, is functionally required for the mutant angiogenic phenotypes.

Furthermore, besides analyzing Gcn2 and Perk, we also used the same approach to examine whether the two other eIF2α kinases, Hri and Pkr, could play an important role in the angiogenic phenotypes of the *tars*^−/^^−^ embryos. Efficiencies of the morpholino-mediated knockdown of Hri and Pkr were validated by the GFP-fusion reporter assay (Supplementary Fig. S2c, d). The results showed that knockdown of Hri or Pkr could not rescue the angiogenesis in the *tars*^−/^^−^ embryos (Supplementary Fig. S7a, b). These results suggest that Hri and Pkr either cannot be activated in this stress condition, or their activities, if any, cannot be transduced to the angiogenesis-regulating mechanisms. Indeed, unlike Gcn2 and Perk as global regulators of cellular stresses, Hri and Pkr exert relatively restricted functions in erythroid cells and immune systems, respectively^6^. Thus, the direct comparison of the four eIF2α kinases in the same system further confirms the necessary and sufficient role of the Gcn2-mediated AAR in *tars*-deficiency-induced angiogenesis.

### The *tars*-deficiency-induced angiogenesis is dependent on the Atf4-Vegfα axis and the ribosome re-initiation mechanism of Atf4 translational regulation

Considering that a possible mechanism underlying the *tars*-deficiency-induced angiogenesis is that the *tars* deficiency may decrease the global protein translation level, we first determined this effect by the O-propargyl-puromycin (OP-puro) incorporation assay^54,55^, in which the OP-puro is incorporated into the nascent proteins in vivo and then can be measured by flow cytometry. Inhibition of protein translation by cycloheximide (CHX) was used as a control in detecting the different OP-puro incorporation levels (Supplementary Fig. S8a). Our results showed that the *tars* deficiency caused a very slight decrease in the protein translation level (Supplementary Fig. S8b). However, inhibition of protein translation with CHX in zebrafish embryos failed to induce increased branches of blood vessels (data not shown), which is consistent with several previous studies involving similar experiments^56–58^. Thus, these results suggest that the angiogenic phenotypes in the *tars* mutants are not caused by the decrease in global protein translation.

To further delineate the role of the downstream portion of the AAR pathway in the induced angiogenesis by *tars* deficiency, we performed morpholino-mediated knockdown of several major factors downstream of eIF2α, including Atf4 and Vegfα, in the *tars*^−/^^−^ and sibling embryos. Meanwhile, as technical complements, a CRISPR interference (CRISPRi)^59^ of Atf4 and a pharmacological inhibition of Vegfα were also performed. The morpholino-mediated knockdown and CRISPRi of Atf4 (encoded by the *atf4a* and *atf4b* homologous genes in zebrafish) both rescued the mutant angiogenic phenotypes (Supplementary Fig. S9). Although zebrafish Vegfα is also encoded by two homologous genes, namely *vegfaa* and *vegfab*, it has been known that only *vegfaa* plays an essential role in regulating embryonic angiogenesis^60^. Our *vegfaa* morpholino-injected embryos recapitulated the phenotype of the *vegfaa* knockout line^60^, as they showed inhibition of ISV growth, which was dependent on the dosage of *vegfaa* morpholino (Supplementary Fig. S10a). Nonetheless, injection of a relatively lower dosage of *vegfaa* morpholino, while incapable of affecting ISV growth, could reduce the ectopic branch points per ISV in the *tars* mutant embryos (Supplementary Fig. S10b). Consistent with these results, the pharmacological inhibition of the Vegf receptor by the chemical SU5416 (also known as Semaxanib)^61^ also led to a dramatic rescue of the mutant phenotypes (Supplementary Fig. S10c). Thus, these results collectively suggest that the Atf4-Vegfα axis acts as a major effector of AAR in the regulation of angiogenesis.

**Fig. 5:**
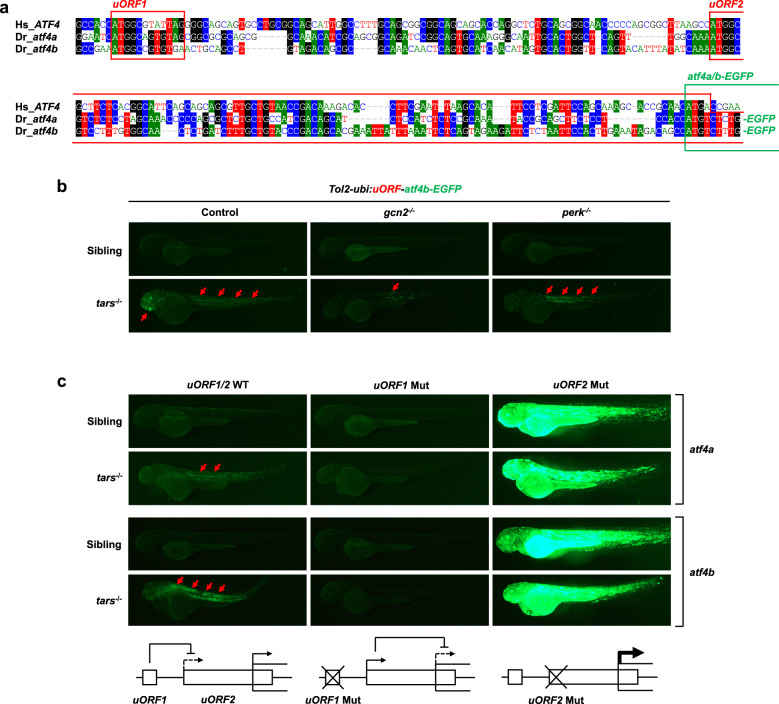
The *tars*-deficiency-induced angiogenesis is dependent on the ribosome re-initiation mechanism of Atf4 translational regulation. **a** An alignment of the sequences of the translational regulatory regions of human *ATF4* and zebrafish *atf4a* and *atf4b*, showing the highly conserved upstream open reading frames (*uORF1* and *uORF2*; red boxes) and coding ORFs (green box). These regions of zebrafish *atf4a* and *atf4b* were cloned into the reporter vectors, and their coding ORFs were fused with the ORF of *EGFP*. **b** Fluorescence microscopy images showing the enhanced expression (i.e., translation) levels of the *atf4a*- and *atf4b-EGFP* fusion ORFs (red arrows) in the truck and head of *tars*^−/^^−^ embryos, which were inhibited by the knockout of *gcn2*, but not *perk*. The Tol2 transposase-based transgenic system containing a *ubiquitin* promoter (*ubi:*) was used to drive the expression of the reporters. **c** Mutation analysis of the zebrafish *atf4a* and *atf4b* uORFs for their eIF2α phosphorylation-dependent translational regulation. The start codon (ATG) of *uORF1* or *uORF2* was mutated into AGG. The expression levels of the WT and *uORF1*- and *uORF2*-mutated (Mut) *atf4a*- and *atf4b-EGFP* reporters were demonstrated by representative fluorescence microscopy images. The lower panels present the working model in which *uORF1* can reduce the usage of the inhibitory *uORF2*, so that the *uORF1* Mut eliminates the AAR-dependent upregulation of *atf4a*/*b* expression, whereas the *uORF2* Mut leads to an extremely high AAR-independent *atf4a*/*b* expression.

We also determined whether the important role of Atf4a/b in this regulation was associated with their translational control mediated by eIF2α phosphorylation. Notably, a comparison of the human *ATF4* and the zebrafish *atf4a* and *atf4b* mRNAs showed evolutionary conservation of their 5′ translational regulatory regions, especially the two upstream ORFs (i.e., the positive-acting *uORF1* and the inhibitory *uORF2*) that have been shown to regulate ATF4 translation through an eIF2α phosphorylation-dependent re-initiation mechanism^18^ (Fig. 5a). To test this mechanism and to assess the translational initiation activities of the *atf4a* and *atf4b* mRNAs in vivo, we employed the uORF reporter assay^18^ by cloning the zebrafish *atf4a* and *atf4b* uORF regions into an EGFP reporter vector and generated Tol2 transposase-mediated transgenic zebrafish^62^. The results indicated that the Atf4a/b translation levels were indeed enhanced in the *tars*^−/^^−^ embryos relative to the siblings and that this enhancement could be dramatically inhibited by the knockout of *gcn2*, but not *perk* (Fig. 5b). Similarly, the Gcn2 inhibitor GCN2-IN-1 could also exert this effect (Supplementary Fig. S11a, b). The notable somite/muscle-enriched expression pattern of the reporter in the trunk, as well as those in the brain, are reminiscent of the expression pattern of *tars* in the developing trunk and brain of the embryos at these stages^63^. Furthermore, this pattern may also be relevant to the previously established important role of the somite-expressed Vegfa^64^ in regulating angiogenesis (and hematopoiesis occurring in the arteries) possibly through paracrine and concentration gradient mechanisms^58,65–67^. Lastly, as a validation of the regulatory mechanism, mutation of the start codon (ATG) to AGG in *uORF1* inhibited the AAR-dependent *atf4a/b-EGFP* expression, likely because of the increased usage of the inhibitory *uORF2* (Fig. 5c), and the ATG-to-AGG mutation of uORF2 indeed led to a high level of AAR-independent *atf4a/b-EGFP* expression (Fig. 5c). Thus, these results provide important evidence and mechanistic explanation for the role of the downstream events of AAR in the *tars*-deficiency-induced angiogenesis.

## Discussion

**Fig. 6:**
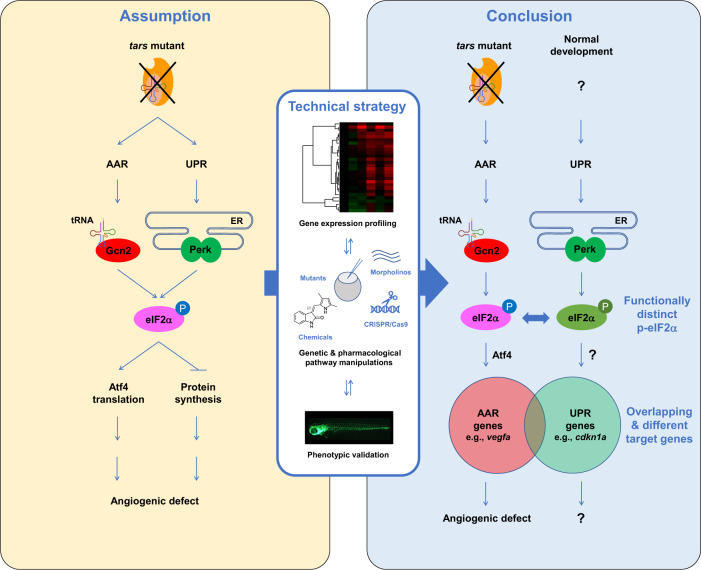
A summary of the study and the working model for the selective and competitive functions of AAR and UPR pathways. The close interconnection of the AAR and UPR pathways makes it difficult to distinguish between them, therefore the angiogenesis induced by aaRS deficiency has been assumptively attributed to activation of both pathways. While UPR has been addressed in previous studies, it is unclear whether AAR is also activated and whether both are required in this process (left panel). We established that these highly interconnected pathways can be distinguished in the herein-generated zebrafish angiogenic model that harbors a *tars* mutation, by using an approach combining systematic gene expression profiling and quantitative phenotypic analysis upon a variety of genetic and pharmacological manipulations of these pathways (middle panel). We found that AAR, but not UPR, is activated and is functionally required for the angiogenic phenotypes in the *tars* mutants (right panel). Notably, while Perk-mediated UPR is inactive in the *tars* mutants, it plays an important role in normal development; however, the function of Perk is overwhelmed by Gcn2 in the stress condition, through competing for phosphorylation of their shared target, eIF2α. The phosphorylated eIF2α (p-eIF2α) by Gcn2 and Perk can be distinguished by the cells/organisms (therefore illustrated in different colors) and thus regulate the partially overlapped AAR- and UPR-associated genes. The question marks denote the possible cause, mechanism, and functional consequence of UPR, which should be different from those of AAR as addressed in this study.

In this study, we investigated a means to specify different roles of AAR and UPR in stress-induced angiogenesis. Our results demonstrated that, despite being closely interconnected and even sharing common downstream targets, AAR and UPR can be activated independently under distinct conditions and their downstream signals can be recognized and transduced differentially in regulating cellular functions such as angiogenesis, which is clearly demonstrated by the dramatically different transcriptome profiles and phenotypes (Fig. 6). This notion reflects the specificity and efficiency of multiple stress response pathways that are evolved integrally to enable the organism to sense and respond precisely to different types of stresses^21^. This study also provides an example of combining systematic transcriptome profiling and phenotypic validations to distinguish the activities of such interconnected pathways (Fig. 6). Clarification of the mechanisms shall advance our understanding of how the organisms respond to diverse stresses and how the abnormalities in these regulatory machinery cause cellular stress-related diseases such as cancer, diabetes, cardiovascular, and immune disorders^1,68–71^.

Tars belong to the family of aaRSs that catalyze the ligation of the 20 amino acids (each by specific aaRSs, albeit separately in cytoplasm and mitochondria) to their cognate tRNAs, thereby determining the fidelity of protein translation^24,25,72^. While mutations in the aaRS genes have been directly implicated in a broad spectrum of human genetic diseases, it remains a significant challenge to understand the underlying mechanism especially of how these basic translation regulators exert tissue-specific effects in these diseases^73–75^. In this study, we observed that the deficiency of *tars* in zebrafish causes angiogenic abnormalities which are dependent on the Tars aminoacylation activity, and, based on this angiogenic model, we identified that AAR instead of UPR plays a dominant role in these angiogenic phenotypes. Regarding the mechanism by which the *tars* deficiency activates AAR, it is notable that the deficiency of an aaRS causes accumulation of uncharged tRNAs, which mimics amino acid deprivation^24,76^, and that the uncharged tRNAs can directly bind to and stimulate Gcn2 kinase activity on eIF2α^29,30^. Thus, the herein specified Tars-Gcn2-eIF2α axis represents a compelling logic to the mechanisms underlying the angiogenesis induced by *tars* deficiency through AAR.

In contrast to the important role of AAR in the *tars*-deficiency-induced angiogenesis, the Perk-mediated UPR pathway shows a very subtle function under this stress condition. Nonetheless, our results of gene expression profiling and knockdown/knockout experiments suggest that Perk and the Perk-mediated UPR are actually active and play an important role in normal development. This finding suggests the importance of understanding the role of stress in the context of the development of living beings, because normal development may require stress and, thus, these “normal” and “stress” conditions may not necessarily be opposite. Indeed, Perk is responsible for the majority of the eIF2α phosphorylation in homeostatic states, whereas Gcn2 only brings a seemingly modest increase beyond this level in the stress condition upon loss of *tars*. It is interesting that such a modest increase of eIF2α phosphorylation exerts strong cellular effects, and that knockdown/knockout of Gcn2 or Perk in this condition, though both can reduce eIF2α phosphorylation to a comparable extend, shows dramatically different phenotypes and targeted gene expression profiles. These observations suggest that there could be a partial switchover of kinases to phosphorylate the eIF2α, and that the cell/organism should be able to distinguish the different phosphorylated eIF2α molecules catalyzed by different kinases. It thus would be interesting to further investigate the mechanism of how the Gcn2- and Perk-catalyzed eIF2α phosphorylation functions differentially. Based on the herein-demonstrated mutual competition between Gnc2 and Perk to phosphorylate eIF2α and the partial switchover of the substrates between these two kinases, it is conceivable that a different subcellular localization of Gcn2 and Perk, as well as their corresponding eIF2α substrates, may enable the selective translation of different groups mRNAs into proteins, which in turn could regulate different gene expression programs. Supportive facts for this mechanism include that Perk is a transmembrane kinase located on ER and, upon UPR/ER-stress, it may further accumulate into specialized subcellular compartments, such as ER-derived quality control compartment^77,78^, whereas Gcn2 is relatively free but can be recruited to the stalled ribosomes through direct interaction with the ribosomal P-stalk proteins in AAR conditions^31,32^. Furthermore, there could also be other mechanisms, such as possible involvement of the two protein phosphatase 1 (PP1) complexes (PP1•GADD34 and PP1•CReP) that dephosphorylate eIF2α and thus antagonize the kinases^5,6^.

The zebrafish aaRS genes are highly conserved with those of human. It has been established that the human aaRS family consists of 38 genes, which encode 18 cytoplasmic, 17 mitochondrial and 3 bifunctional aaRS enzymes^73,79^. Notably, besides the cytoplasmic TARS and the mitochondrial TARS2, TARSL2 is a newly identified cytoplasmic TARS homolog that possesses tRNA aminoacylation and editing activities and locates in not only cytoplasm but also nucleus^79–82^. Similarly, zebrafish also contains the *tarsl2* gene (data not shown), although its function has been unknown. Therefore, it would be interesting to investigate whether the *tars* deficiency could be at least partially compensated by *tarsl2*. Further, among all the aaRSs, there should also be a balanced ratio, as well as the ratio with cognate tRNAs, to regulate the fidelity of protein translation^24,72^. In this regard, some aaRSs may be involved in regulating similar biological processes. Indeed, besides Tars, several other aaRSs, including Hars, Iars, Sars, and Wars2, have also been shown to regulate angiogenesis^56–58,63,83–89^. Therefore, the angiogenic regulatory functions and the herein identified underlying mechanisms may be shared by many, though probably not all, aaRSs.

In conclusion, although AAR and UPR are evolved to sense and respond to different types of stresses, their close interconnection makes it difficult to distinguish between them, and this situation has led to biased or over-simplified assumptions on the mechanisms of regulation of angiogenesis by AAR and/or UPR. In this study, by means of a systematic gene expression profiling in combination with a variety of genetic and pharmacological manipulations of these pathways and phenotypic validations in zebrafish models, we have been able to specify the different roles of AAR and UPR in stress-induced angiogenesis (Fig. 6). Notably, this technical strategy should also be readily applicable in an analysis of cells/tissues cultured in vitro, as well as in patient-derived xenograft models, to better define the activities of different stress response pathways. Given the critical importance of these pathways in the pathogenesis of various human diseases and as potential therapeutic targets, it thus will be possible, and of great significance, to clearly distinguish and specifically manipulate these pathways in patient samples using this strategy, which may facilitate identification of stress-related biomarkers and therapeutic strategies.

## Materials and methods

### Zebrafish strains

The zebrafish strain *Tübingen* (ZFIN ID: ZDB-GENO-990623-3) and the transgenic zebrafish line Tg(*flk1:EGFP*) (ZFIN ID: ZDB-ALT-050916-14)^35^ were maintained in system water under standard conditions at 28.5 °C with a photoperiod of 12 h of light and 12 h of dark. Both males and females were used. Developmental stages of the embryos were indicated in the figures and legends. The embryos were grown in egg water containing 60 µg/mL sea salt and 0.2% methylene blue at 28.5 °C. *N*-Phenylthiourea (PTU; Sigma-Aldrich; 0.045%) was used to prevent pigmentation. All experiments using animals were approved by the Committee of Animal Use for Research at Shanghai Jiao Tong University School of Medicine.

### Generation of the *tars* mutant zebrafish

The zebrafish mutant line harboring a loss-of-function mutation in the *tars* gene (ZFIN ID: ZDB-GENE-041010-218) was identified in a forward genetics screening of hematopoietic/angiogenetic mutants through the treatment of ENU (Sigma) as previously described^33,34^. Whole exosome sequencing was performed to identify the mutated gene. In brief, we first captured the exome regions with the SureSelect non-human Exomes (SSXT Zebrafish All Exon, 16rxn) kit (Agilent Technologies) and then performed high-throughput DNA sequencing using the Illumina Genome Analyzer IIx (GAIIx) according to the manufacturer’s protocols. The PCR primers for genotyping are listed in Supplementary Table S5. The *tars* mutant line was maintained as heterozygous because the homozygous embryos were lethal at around 6 dpf.

### Generation of the *gcn2* and *perk* knockout zebrafish by CRISPR/Cas9 genome editing

For a generation of *gcn2* and *perk* knockout zebrafish, guide RNAs (gRNAs) were designed using ZiFiT Targeter software (http://zifit.partners.org/ZiFiT/CSquare9Nuclease.aspx). The gRNAs of *gcn2* (GGACGCTCTGCCGGCGCGGG; targeting the PKD domain) and *perk* (GGACTCATCATGCATGTGTG; targeting the catalytic domain) were transcribed in vitro and injected into embryos of the one-cell stage with 200 ng/mL Cas9 protein (New England Biolabs, M0646T) following the method as described previously^90^. Primers for genotyping for *perk* and *gcn2* were listed in Supplementary Table S5. The CRISPRi assay was similarly designed and performed with the Tg(*flk1:EGFP*) line, and the chimera F0 embryos were validated by sequencing and subjected to phenotypic analysis (Supplementary Fig. S9).

### Visualization and quantitative analysis of the angiogenic phenotypes

Live embryos were anesthetized with 0.03% ethyl 3-aminobenzoate methanesulfonate salt (Tricaine) (Sigma; A5040) and mounted in 1% low melting agarose (Sangon Biotech; A600015). Fluorescence images were captured with a scanning confocal microscope (Olympus; FV1000) processed with Image-Pro Plus 6.0 (Media Cybernetics). To quantify the angiogenic phenotypes in the trunk, the blood vessels in the region encompassing 8–13 ISVs anterior to the end of yolk extension were analyzed, and the number of ectopic branching points per ISV^37^ was calculated statistically.

### Morpholino-mediated gene knockdown

All morpholino oligonucleotides were synthesized by Gene Tools. The sequences of the morpholinos that were designed for the first time and used in this study, including *gcn2* MO, *hri* MO, *pkr* MO, *eif2s1a* MO, *eif2s1b* MO, and *atf4b* MO, were presented in Supplementary Fig. S2 and Table S5. Besides, several previously reported morpholinos, including *tars* MO^4^, *perk* MO^34^, *atf4a* MO^4^, and *vegfaa* MO^40^ were also employed in this study; their sequences were also shown in Supplementary Table S5. All morpholinos were injected into the embryos at the one-cell stage.

### mRNA injection for phenotype rescue

Zebrafish *tars* cDNA was amplified by RT-PCR from zebrafish embryo samples and inserted into the pCS2^+^ vector that contains a FLAG tag. Mutagenesis was performed with the QuikChange Site-Directed Mutagenesis Kit (Agilent Technologies; 200519). All capped mRNAs were transcribed in vitro with the SP6 mMESSAGE mMACHINE Transcription Kit (Thermo Fisher Scientific; AM1340) and purified with the NucAway Spin Columns (Thermo Fisher Scientific; AM10070). The purified mRNAs were diluted to 100 ng/µL for microinjection into the embryos.

### Tars protein purification and tRNA in vitro transcription

Zebrafish *tars*-WT, -R459A, and -H473A cDNA were amplified from the pCS2^+^ vector mentioned above and inserted into the pET28a plasmid. Tars and their mutant proteins were purified according to the methods reported previously^91^. Transcription of zebrafish tRNA^Thr^ in vitro, including tRNA^Thr^(AGU), was performed as previously described^92^.

### Aminoacylation assay

According to a previously described procedure^91^, time course curves of aminoacylation by zebrafish Tars-WT, -R459A, and -H473A proteins were determined at 37 °C in a reaction mixture (30 μL) containing 60 μM Tris-HCl (pH 7.5), 10 mM MgCl_2_, 5 mM DTT, 2.5 mM ATP, 114 μM [^14^C] l-Threonine and 200 nM protein with 5 μM of tRNA^thr^.

### Northern blot

Total RNAs of zebrafish embryos were isolated using Trizol reagent, and 5 μg of total RNAs were electrophoresed through 15% polyacrylamide-8 M urea gel in Tris-borate-EDTA buffer at room temperature under 150 V for 1.5 h. For the aminoacylation assays, total RNAs were extracted and resolved with 0.1 M NaAc (pH 5.2). To separate the charged and uncharged tRNAs, 5 μg of total RNAs were electrophoresed through an acidic (pH 5.2) 10% polyacrylamide-8 M urea gel at 4 °C under 18 W for 16 h. The RNAs were then transferred onto a positively charged nylon membrane at 4 °C under 250 mA for 30 min. After UV-crosslink (8000 × 100 J/cm^2^), the membrane was pre-blocked with pre-hybridization solution (4× SSC, 1 M Na_2_HPO_4_, 7% SDS, 1.5× denhardt solution, 0.4 mg/L fish sperm DNA) at 55 °C for 2 h. The membrane was hybridized with digoxin (DIG)-labeled probes for specific tRNAs and 5S rRNA at 55 °C overnight and the probe sequences were listed in Supplementary Table S5. Because of the high similar sequences between tRNA^Thr^(AGU) and tRNA^Thr^(CGU), one probe was designed to define both of them, and the probe was named tRNA^Thr^(AGU/CGU). The membrane was washed with washing buffer (0.1 M maleic acid, 0.15 M NaCl, pH7.5) twice for 15 min and blocked with 1× blocked reagent (0.1 M maleic acid, 0.15 M NaCl, 10% blocking reagent, pH7.5) for 30 min at room temperature. Then, it was incubated with anti-AP buffer (1× blocked reagent, 1:10,000 anti-digoxigenin-AP) for 1 h and washed twice. Finally, the membrane was treated with the CDP-Star, and imaged by the Amersham imager 680 system (GE, CA, USA).

### RNA-seq and bioinformatics analysis

Total RNA of the embryos at 36 hpf was extracted using the TRIzol reagent (Ambion; 15596018). RNA-seq libraries were constructed using the NEBNext UltraTM RNA Library Prep Kit for Illumna (New England Biolabs; E7530) and sequenced on an Illumina Hiseq4000 platform. The zebrafish reference genome (Danio_rerio.GRCz10.dna.toplevel.fa) and gene model annotation (Danio_rerio.GRCz10.91.chr.gtf.gz) files were downloaded from ENSEMBL. Hisat2 (version 2.0.5)^93^ was used to build the index of the reference genome and to align the clean reads to the reference genome. The read numbers mapped to each gene were counted and the FPKM of each gene was calculated with HTSeq (version 0.9.1)^94^. The human homologs of zebrafish genes were determined with the HomoloGene database (https://www.ncbi.nlm.nih.gov/homologene) and the reciprocal best hits strategy as described previously^95,96^. Hierarchical clustering and PCA were performed with Cluster (version 3.0)^97^ and the heatmap was presented with Java TreeView (version 1.1.6r4)^98^. GSEA^99^ was performed to identify regulated signaling pathways and to interpret gene expression patterns globally.

### RT-qPCR

RNA was extracted from embryos using TRIzol reagent (Ambion; 15596018). RNA was reverse transcribed using random hexamers and oligo(dT) primers. The SYBR Green Real-Time PCR Master Mix kit (TOYOBO; QPK-201) was used for the qPCR analysis on the ABI Prism 7900HT Sequence Detection System (Applied Biosystems). The relative expression values were normalized against the internal control *β-actin* (*actb1*) gene. Sequences of the PCR primers were listed in Supplementary Table S5.

### Immunoblot

The embryos were deyolked and the proteins were extracted by lysis buffer (50 mM Tris-HCl (pH 7.4), 150 mM NaCl, 1% NP-40, 0.25% sodium deoxycholate, 5 mM EDTA, 10% glycerol and 0.1% Triton X-100) containing appropriate protease inhibitors (Bimake; B14001) and phosphatase inhibitor cocktail (Bimake; B15001). The boiled protein samples were first analyzed by SDS-PAGE and Coomassie blue staining as a preliminary normalization^100^. Immunoblot analysis was performed with antibodies including anti-phospho-eIF2α (Ser51) (D9G8) XP rabbit monoclonal antibody (mAb) (Cell Signaling Technology; 3398), anti-eIF2α antibody (Cell Signaling Technology; 9722), anti-phospho-p70 S6 Kinase (Thr389) (108D2) Rabbit mAb (Cell Signaling Technology; 9234), anti-p70 S6 Kinase (49D7) Rabbit mAb (Cell Signaling Technology; 2708) and anti-β-actin mouse mAb (YEASEN; 30101ES60).

### Chemical treatment

Dechorionated embryos were treated with 1 μM SU5416 (Merck KGaA; 676487), 20 μM GSK2656157 (MedChemExpress; HY-13820), 10 μM GCN2-IN-1 (MedChemExpress; HY-100877), 10 μM GCN2iB (MedChemExpress; HY-112654), or 50 μM 4-Phenylbutyric acid (4-PBA) (Sigma Aldrich; P21005) from 24 to 54 hpf. To inhibit global protein translation, the embryos were treated with 360 μM CHX (Sigma-Aldrich; C7698). While 4-PBA was dissolved in water, the other chemicals were dissolved in Dimethyl sulfoxide (DMSO) (Sigma-Aldrich; D8418). The solvents, water or DMSO, were used as controls, respectively, and the final concentration of DMSO is 0.1%.

### OP-puro incorporation assay

The embryos, including WT, *tars*^−/−^, and those treated with CHX, were incubated with 5 mM OP-puro in egg water containing 15% DMSO for 25 min at 28.5 °C. As unstained controls, the WT embryos were also incubated with 15% DMSO in egg water in the same condition. The embryos were rinsed with egg water gently 3 times and further grown for 1 h. Then the embryos were deyolked and treated with 1 mg/mL collagenase IV (Gibco; 17104-019) for 30 min at 30 °C. The reaction was stopped with Fetal Bovine Serum (FBS) (Sigma-Aldrich; F2442), and the lysis products were centrifuged at 800× *g* for 5 min at 4 °C. The pellets were resuspended and washed twice with 10% FBS in PBS and filtered through a 40 μm cell-strainer (Falcon; 352235). The cell suspensions were fixed with 4% paraformaldehyde (Sigma-Aldrich; P6148) at 4 °C overnight. The fixed cells were washed with PBS and permeabilized in PBS supplemented with 0.05% Triton X-100 (Sigma-Aldrich; V900502) for 15 min at room temperature, followed by another wash with PBS supplemented with 0.05% Triton X-100 and 3% bovine serum albumin (BSA). Azide-alkyne reaction was performed using Click-iT Cell Reaction Buffer Kit (Life Technologies; C10269) according to the manufacture’s protocols. The azide was conjugated by Tetramethylrhodamine (Life Technologies; T10182) at a final concentration of 5 μM. DAPI (Beyotime Biotechnology; C1002) was also added at 5 μg/mL to stain the cell nucleus. After 30 min of reaction, the cells were washed twice with PBS supplemented with 0.05% Triton X-100 and 3% BSA and were subjected to flow cytometry analysis. For analysis of the “Mean OP-Puro fluorescence”, the fluorescence values per DAPI-positive cell were calculated.

### *atf4a/b* translation reporter assay

The 5′ regions of zebrafish *atf4a* (ZFIN ID: ZDB-GENE-040426-2340) and *atf4b* (ZFIN ID: ZDB-GENE-070928-23) mRNAs, including uORF1, partial uORF2, and the first 9-bp of the coding ORFs, were cloned into the pCS2^+^-EGFP vector to be fused with the *EGFP* ORF. Then, the *uORF-atf4a-EGFP* and *uORF-atf4b-EGFP* cassettes were inserted into the Tol2 transposon vector containing the *ubiquitin* promoter. For Tol2-mediated transient transgenesis, the Tol2 vectors (180 pg) and Tol2 transposase mRNA (90 pg) were injected as previously reported^62^ and the expression of EGFP was visualized with a fluorescence microscope.

### Quantification and statistical analysis

For quantitative angiogenic phenotype analysis, the ectopic branch points per ISV of the *tars*^−/−^ and sibling embryos were quantified, and all the values of each embryo, as well as their means ± SD, were presented as scatter plots in the Figures. For the RT-qPCR experiments, the data were presented as means ± SD of triplicate reactions. A two-tailed Student’s *t-*test was used for these statistical analyses with the GraphPad Prism 6 program, and *P* < 0.05 was considered statistically significant.

## Supplementary information

Supplementary Information

## Data Availability

RNA-seq data are accessible through the Gene Expression Omnibus (GEO) accession code GSE130193.

## References

[1] Carmeliet P, Jain RK (2000). Angiogenesis in cancer and other diseases. Nature.

[2] Longchamp A (2018). Amino acid restriction triggers angiogenesis via GCN2/ATF4 regulation of VEGF and H2S production. Cell.

[3] Binet F, Sapieha P (2015). ER stress and angiogenesis. Cell Metab..

[4] Castranova D (2016). Aminoacyl-transfer RNA synthetase deficiency promotes angiogenesis via the unfolded protein response pathway. Arterioscler. Thromb. Vasc. Biol..

[5] Pakos-Zebrucka K (2016). The integrated stress response. EMBO Rep..

[6] Wek RC (2018). Role of eIF2alpha kinases in translational control and adaptation to cellular stress. Cold Spring Harb. Perspect. Biol..

[7] Dever TE (1992). Phosphorylation of initiation factor 2 alpha by protein kinase GCN2 mediates gene-specific translational control of GCN4 in yeast. Cell.

[8] Berlanga JJ, Santoyo J, De Haro C (1999). Characterization of a mammalian homolog of the GCN2 eukaryotic initiation factor 2alpha kinase. Eur. J. Biochem..

[9] Sood R, Porter AC, Olsen DA, Cavener DR, Wek RC (2000). A mammalian homologue of GCN2 protein kinase important for translational control by phosphorylation of eukaryotic initiation factor-2alpha. Genetics.

[10] Shi Y (1998). Identification and characterization of pancreatic eukaryotic initiation factor 2 alpha-subunit kinase, PEK, involved in translational control. Mol. Cell Biol..

[11] Harding HP, Zhang Y, Ron D (1999). Protein translation and folding are coupled by an endoplasmic-reticulum-resident kinase. Nature.

[12] Ranu RS, London IM (1976). Regulation of protein synthesis in rabbit reticulocyte lysates: purification and initial characterization of the cyclic 3’:5’-AMP independent protein kinase of the heme-regulated translational inhibitor. Proc. Natl Acad. Sci. USA.

[13] Chen JJ (1991). Cloning of the cDNA of the heme-regulated eukaryotic initiation factor 2 alpha (eIF-2 alpha) kinase of rabbit reticulocytes: homology to yeast GCN2 protein kinase and human double-stranded-RNA-dependent eIF-2 alpha kinase. Proc. Natl Acad. Sci. USA.

[14] Farrell PJ, Balkow K, Hunt T, Jackson RJ, Trachsel H (1977). Phosphorylation of initiation factor elF-2 and the control of reticulocyte protein synthesis. Cell.

[15] Meurs E (1990). Molecular cloning and characterization of the human double-stranded RNA-activated protein kinase induced by interferon. Cell.

[16] Ron, D. Translational control in the endoplasmic reticulum stress response. *J. Clin. Invest.* **110**, 1383–1388 (2002). 10.1172/JCI16784 PMC151821 12438433

[17] Harding HP (2003). An integrated stress response regulates amino acid metabolism and resistance to oxidative stress. Mol. Cell.

[18] Vattem KM, Wek RC (2004). Reinitiation involving upstream ORFs regulates ATF4 mRNA translation in mammalian cells. Proc. Natl Acad. Sci. USA.

[19] Kilberg MS, Shan J, Su N (2009). ATF4-dependent transcription mediates signaling of amino acid limitation. Trends Endocrinol. Metab..

[20] Han J, Kaufman RJ (2017). Physiological/pathological ramifications of transcription factors in the unfolded protein response. Genes Dev..

[21] Kultz D (2005). Molecular and evolutionary basis of the cellular stress response. Annu Rev. Physiol..

[22] Rothenburg, S., Georgiadis, M. M. & Wek, R. C. Evolution of eIF2α Kinases: Adapting Translational Control to Diverse Stresses. In: Hernández G, Jagus G, eds. Evolution of the Protein Synthesis Machinery and Its Regulation. Springer, 235–260 (2016).

[23] Dang Do AN, Kimball SR, Cavener DR, Jefferson LS (2009). eIF2alpha kinases GCN2 and PERK modulate transcription and translation of distinct sets of mRNAs in mouse liver. Physiol. Genomics.

[24] Ling J, Reynolds N, Ibba M (2009). Aminoacyl-tRNA synthesis and translational quality control. Annu. Rev. Microbiol..

[25] Schimmel P (2018). The emerging complexity of the tRNA world: mammalian tRNAs beyond protein synthesis. Nat. Rev. Mol. Cell Biol..

[26] Lounsbury KM, Francklyn CS (2016). Aminoacyl-transfer RNA synthetases: connecting nutrient status to angiogenesis through the unfolded protein response. Arterioscler. Thromb. Vasc. Biol..

[27] Guo M, Schimmel P (2013). Essential nontranslational functions of tRNA synthetases. Nat. Chem. Biol..

[28] Lo WS (2014). Human tRNA synthetase catalytic nulls with diverse functions. Science.

[29] Wek SA, Zhu S, Wek RC (1995). The histidyl-tRNA synthetase-related sequence in the eIF-2 alpha protein kinase GCN2 interacts with tRNA and is required for activation in response to starvation for different amino acids. Mol. Cell Biol..

[30] Dong J, Qiu H, Garcia-Barrio M, Anderson J, Hinnebusch AG (2000). Uncharged tRNA activates GCN2 by displacing the protein kinase moiety from a bipartite tRNA-binding domain. Mol. Cell.

[31] Harding HP (2019). The ribosomal P-stalk couples amino acid starvation to GCN2 activation in mammalian cells. Elife.

[32] Inglis AJ (2019). Activation of GCN2 by the ribosomal P-stalk. Proc. Natl Acad. Sci. USA.

[33] Peng X (2015). A point mutation of zebrafish c-cbl gene in the ring finger domain produces a phenotype mimicking human myeloproliferative disease. Leukemia.

[34] Jia XE (2015). Mutation of kri1l causes definitive hematopoiesis failure via PERK-dependent excessive autophagy induction. Cell Res..

[35] Jin SW, Beis D, Mitchell T, Chen JN, Stainier DY (2005). Cellular and molecular analyses of vascular tube and lumen formation in zebrafish. Development.

[36] Jin SW (2007). A transgene-assisted genetic screen identifies essential regulators of vascular development in vertebrate embryos. Dev. Biol..

[37] Villefranc JA (2013). A truncation allele in vascular endothelial growth factor c reveals distinct modes of signaling during lymphatic and vascular development. Development.

[38] Sankaranarayanan R (1999). The structure of threonyl-tRNA synthetase-tRNA(Thr) complex enlightens its repressor activity and reveals an essential zinc ion in the active site. Cell.

[39] Xu PF (2010). Setdb2 restricts dorsal organizer territory and regulates left-right asymmetry through suppressing fgf8 activity. Proc. Natl Acad. Sci. USA.

[40] Nasevicius A, Larson J, Ekker SC (2000). Distinct requirements for zebrafish angiogenesis revealed by a VEGF-A morphant. Yeast.

[41] Krige D (2008). CHR-2797: an antiproliferative aminopeptidase inhibitor that leads to amino acid deprivation in human leukemic cells. Cancer Res..

[42] Peng T, Golub TR, Sabatini DM (2002). The immunosuppressant rapamycin mimics a starvation-like signal distinct from amino acid and glucose deprivation. Mol. Cell Biol..

[43] Jousse C, Bruhat A, Ferrara M, Fafournoux P (1998). Physiological concentration of amino acids regulates insulin-like-growth-factor-binding protein 1 expression. Biochem. J..

[44] Vazquez de Aldana CR, Wek RC, Segundo PS, Truesdell AG, Hinnebusch AG (1994). Multicopy tRNA genes functionally suppress mutations in yeast eIF-2 alpha kinase GCN2: evidence for separate pathways coupling GCN4 expression to unchanged tRNA. Mol. Cell Biol..

[45] Qiu H (2000). Defects in tRNA processing and nuclear export induce GCN4 translation independently of phosphorylation of the alpha subunit of eukaryotic translation initiation factor 2. Mol. Cell Biol..

[46] Nakamura A, Kimura H (2017). A new role of GCN2 in the nucleolus. Biochem. Biophys. Res. Commun..

[47] Li Z, Vuong JK, Zhang M, Stork C, Zheng S (2017). Inhibition of nonsense-mediated RNA decay by ER stress. RNA.

[48] Masson GR (2019). Towards a model of GCN2 activation. Biochem. Soc. Trans..

[49] Brazeau JF, Rosse G (2014). Triazolo[4,5-d]pyrimidine derivatives as inhibitors of GCN2. ACS Med. Chem. Lett..

[50] Nakamura A (2018). Inhibition of GCN2 sensitizes ASNS-low cancer cells to asparaginase by disrupting the amino acid response. Proc. Natl Acad. Sci. USA.

[51] Atkins C (2013). Characterization of a novel PERK kinase inhibitor with antitumor and antiangiogenic activity. Cancer Res..

[52] Rubenstein, R. C., Egan, M. E. & Zeitlin, P. L. In vitro pharmacologic restoration of CFTR-mediated chloride transport with sodium 4-phenylbutyrate in cystic fibrosis epithelial cells containing delta F508-CFTR. *J. Clin. Invest.* **100**, 2457–2465 (1997). 10.1172/JCI119788 PMC508446 9366560

[53] Kubota K (2006). Suppressive effects of 4-phenylbutyrate on the aggregation of Pael receptors and endoplasmic reticulum stress. J. Neurochem..

[54] Liu J, Xu Y, Stoleru D, Salic A (2012). Imaging protein synthesis in cells and tissues with an alkyne analog of puromycin. Proc. Natl Acad. Sci. USA.

[55] Signer RA, Magee JA, Salic A, Morrison SJ (2014). Haematopoietic stem cells require a highly regulated protein synthesis rate. Nature.

[56] Fukui H, Hanaoka R, Kawahara A (2009). Noncanonical activity of seryl-tRNA synthetase is involved in vascular development. Circ. Res..

[57] Herzog W, Muller K, Huisken J, Stainier DY (2009). Genetic evidence for a noncanonical function of seryl-tRNA synthetase in vascular development. Circ. Res..

[58] Ni R, Luo L (2018). A noncanonical function of histidyl-tRNA synthetase: inhibition of vascular hyperbranching during zebrafish development. FEBS Open Bio.

[59] Stainier DYR (2017). Guidelines for morpholino use in zebrafish. PLoS Genet..

[60] Jin D (2017). Vegfa signaling regulates diverse artery/vein formation in vertebrate vasculatures. J. Genet. Genomics.

[61] Bold G (2000). New anilinophthalazines as potent and orally well absorbed inhibitors of the VEGF receptor tyrosine kinases useful as antagonists of tumor-driven angiogenesis. J. Med. Chem..

[62] Gao L (2015). TopBP1 governs hematopoietic stem/progenitor cells survival in zebrafish definitive hematopoiesis. PLoS Genet..

[63] Jeong SJ (2019). A threonyl-tRNA synthetase-mediated translation initiation machinery. Nat. Commun..

[64] Liang D (1998). Cloning and characterization of vascular endothelial growth factor (VEGF) from zebrafish, *Danio rerio*. Biochim. Biophys. Acta.

[65] Lawson ND, Vogel AM, Weinstein BM (2002). Sonic hedgehog and vascular endothelial growth factor act upstream of the Notch pathway during arterial endothelial differentiation. Dev. Cell.

[66] Carroll KJ (2014). Estrogen defines the dorsal-ventral limit of VEGF regulation to specify the location of the hemogenic endothelial niche. Dev. Cell.

[67] Genthe JR, Clements WK (2017). R-spondin 1 is required for specification of hematopoietic stem cells through Wnt16 and Vegfa signaling pathways. Development.

[68] Hotamisligil GS, Davis RJ (2016). Cell signaling and stress responses. Cold Spring Harb. Perspect. Biol..

[69] Tahmasebi S, Khoutorsky A, Mathews MB, Sonenberg N (2018). Translation deregulation in human disease. Nat. Rev. Mol. Cell Biol..

[70] Cao Y (2013). Angiogenesis and vascular functions in modulation of obesity, adipose metabolism, and insulin sensitivity. Cell Metab..

[71] Donato AJ, Machin DR, Lesniewski LA (2018). Mechanisms of dysfunction in the aging vasculature and role in age-related disease. Circ. Res..

[72] Mohler K, Ibba M (2017). Translational fidelity and mistranslation in the cellular response to stress. Nat. Microbiol..

[73] Antonellis A, Green ED (2008). The role of aminoacyl-tRNA synthetases in genetic diseases. Annu. Rev. Genomics Hum. Genet..

[74] Meyer-Schuman R, Antonellis A (2017). Emerging mechanisms of aminoacyl-tRNA synthetase mutations in recessive and dominant human disease. Hum. Mol. Genet..

[75] Park SG, Schimmel P, Kim S (2008). Aminoacyl tRNA synthetases and their connections to disease. Proc. Natl Acad. Sci. USA.

[76] Hao S (2005). Uncharged tRNA and sensing of amino acid deficiency in mammalian piriform cortex. Science.

[77] Kondratyev M, Avezov E, Shenkman M, Groisman B, Lederkremer GZ (2007). PERK-dependent compartmentalization of ERAD and unfolded protein response machineries during ER stress. Exp. Cell Res..

[78] Leitman J (2014). Herp coordinates compartmentalization and recruitment of HRD1 and misfolded proteins for ERAD. Mol. Biol. Cell.

[79] Chen Y (2018). A threonyl-tRNA synthetase-like protein has tRNA aminoacylation and editing activities. Nucleic Acids Res..

[80] Kim K (2013). Reinvestigation of aminoacyl-tRNA synthetase core complex by affinity purification-mass spectrometry reveals TARSL2 as a potential member of the complex. PLoS ONE.

[81] Park SJ, Ahn HS, Kim JS, Lee C (2015). Evaluation of multi-tRNA synthetase complex by multiple reaction monitoring mass spectrometry coupled with size exclusion chromatography. PLoS ONE.

[82] Zhou XL (2019). Newly acquired N-terminal extension targets threonyl-tRNA synthetase-like protein into the multiple tRNA synthetase complex. Nucleic Acids Res..

[83] Xu X (2012). Unique domain appended to vertebrate tRNA synthetase is essential for vascular development. Nat. Commun..

[84] Lin CY (2013). MiR-1 and miR-206 target different genes to have opposing roles during angiogenesis in zebrafish embryos. Nat. Commun..

[85] Shi Y (2014). tRNA synthetase counteracts c-Myc to develop functional vasculature. Elife.

[86] Mirando AC (2015). Aminoacyl-tRNA synthetase dependent angiogenesis revealed by a bioengineered macrolide inhibitor. Sci. Rep..

[87] Cao Z, Wang H, Mao X, Luo L (2016). Noncanonical function of threonyl-tRNA synthetase regulates vascular development in zebrafish. Biochem. Biophys. Res. Commun..

[88] Wang M (2016). Wars2 is a determinant of angiogenesis. Nat. Commun..

[89] Fu CY, Wang PC, Tsai HJ (2017). Competitive binding between Seryl-tRNA synthetase/YY1 complex and NFKB1 at the distal segment results in differential regulation of human vegfa promoter activity during angiogenesis. Nucleic Acids Res..

[90] Liu DJ (2020). setd2 knockout zebrafish is viable and fertile: differential and developmental stress-related requirements for Setd2 and histone H3K36 trimethylation in different vertebrate animals. Cell Discov..

[91] Zhou XL, Zhu B, Wang ED (2008). The CP2 domain of leucyl-tRNA synthetase is crucial for amino acid activation and post-transfer editing. J. Biol. Chem..

[92] Zeng QY (2019). The G3-U70-independent tRNA recognition by human mitochondrial alanyl-tRNA synthetase. Nucleic Acids Res..

[93] Pertea M, Kim D, Pertea GM, Leek JT, Salzberg SL (2016). Transcript-level expression analysis of RNA-seq experiments with HISAT, StringTie and Ballgown. Nat. Protoc..

[94] Anders S, Pyl PT, Huber W (2015). HTSeq-a Python framework to work with high-throughput sequencing data. Bioinformatics.

[95] Song HD (2004). Hematopoietic gene expression profile in zebrafish kidney marrow. Proc. Natl Acad. Sci. USA.

[96] Sun XJ (2008). Genome-wide survey and developmental expression mapping of zebrafish SET domain-containing genes. PLoS ONE.

[97] Eisen MB, Spellman PT, Brown PO, Botstein D (1998). Cluster analysis and display of genome-wide expression patterns. Proc. Natl Acad. Sci. USA.

[98] Saldanha AJ (2004). Java Treeview-extensible visualization of microarray data. Bioinformatics.

[99] Subramanian A (2005). Gene set enrichment analysis: a knowledge-based approach for interpreting genome-wide expression profiles. Proc. Natl Acad. Sci. USA.

[100] Zhang MM (2019). Destabilization of AETFC through C/EBPalpha-mediated repression of LYL1 contributes to t(8;21) leukemic cell differentiation. Leukemia.

