# Supplementary material for: Selective and competitive functions of the AAR and UPR pathways in stress-induced angiogenesis
Source: Cell Discov. 2021 Oct 26;7:98. doi: 10.1038/s41421-021-00332-8 (PMC8547220; doi:10.1038/s41421-021-00332-8)
Supplement: Supplementary file 1 — Supplementary Information [file 41421_2021_332_MOESM1_ESM.pdf]

Zhang et al., Selective and competitive functions of the AAR and UPR pathways in stress-induced angiogenesis

Supplementary Information, containing 11 Supplementary Figures, 5 Supplementary Tables and 6 Supplementary References.

Supplementary Figures

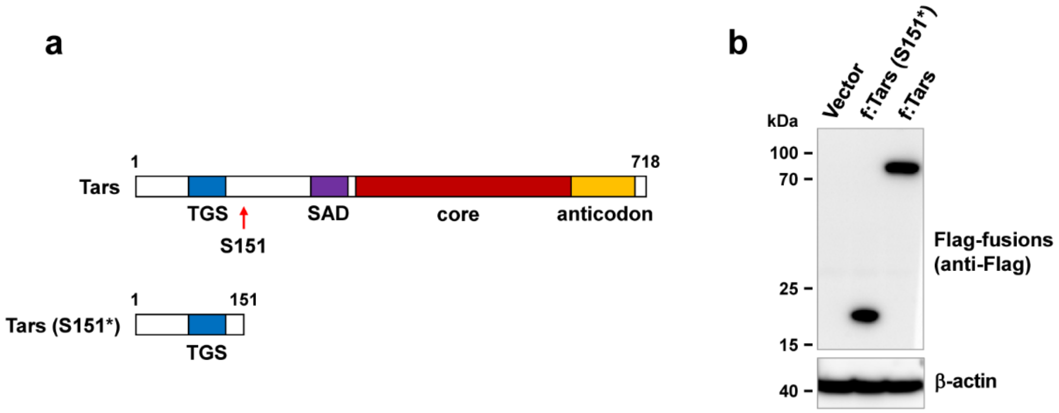

Supplementary Fig. S1 The mutated *tars* gene can only encode a truncated protein. a Domain architecture of the wild-type (WT) and mutant (S151*) Tars proteins. **b** Ectopic expression and immunoblot analysis of tars WT and mutant proteins fused with Flag (f:) tags at the N-terminus. Full-length WT and mutant *tars* cDNAs were cloned from the WT and mutant embryos. The proteins were expression in NIH 3T3 cells, and β-actin was used as a loading control.

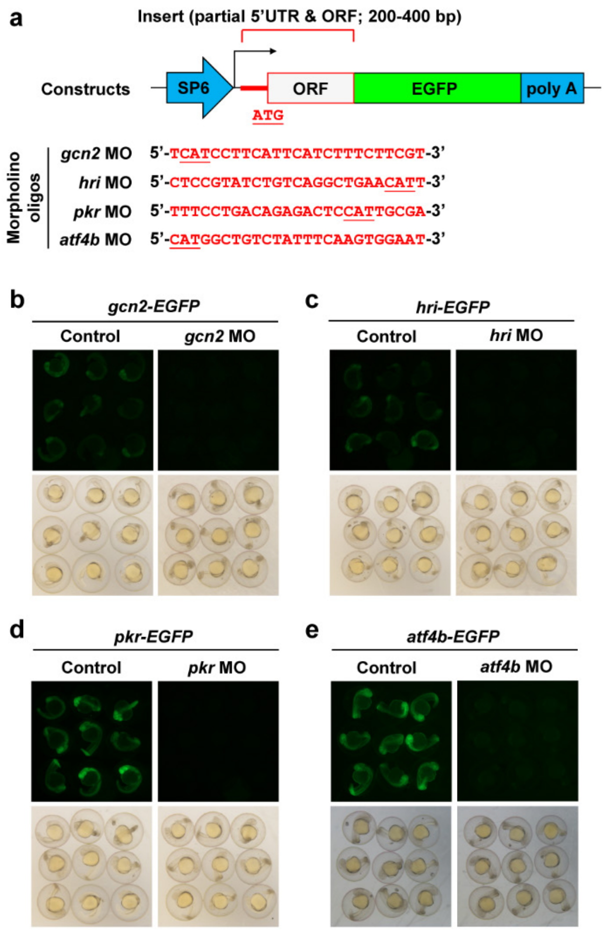

Supplementary Fig. S2 Verification of the efficiency of the morpholinos that were designed for the first time and used in this study. a Targeting and validation strategies and the sequences of the morpholino oligos (MOs), which are designed to inhibit protein translation. The partial 5'UTRs and ORFs of the target genes containing morpholino target sequences are fused in frame with EGFP followed by a polyA site. The start codon (ATG) in the targeted ORFs and its complementary sequence (CAT) in the morpholinos are underlined. Other morpholinos used in this study have been described previously and their sequences and references are listed in Supplementary Table S5. **b–e** Fluorescence microscopy (*top*) and bright field images (*bottom*) of representative embryos injected with indicated EGFP-fusion mRNAs with or without morpholinos. Note that these morpholinos can significantly inhibit the expression of EGFP, indicative of an effective knockdown.

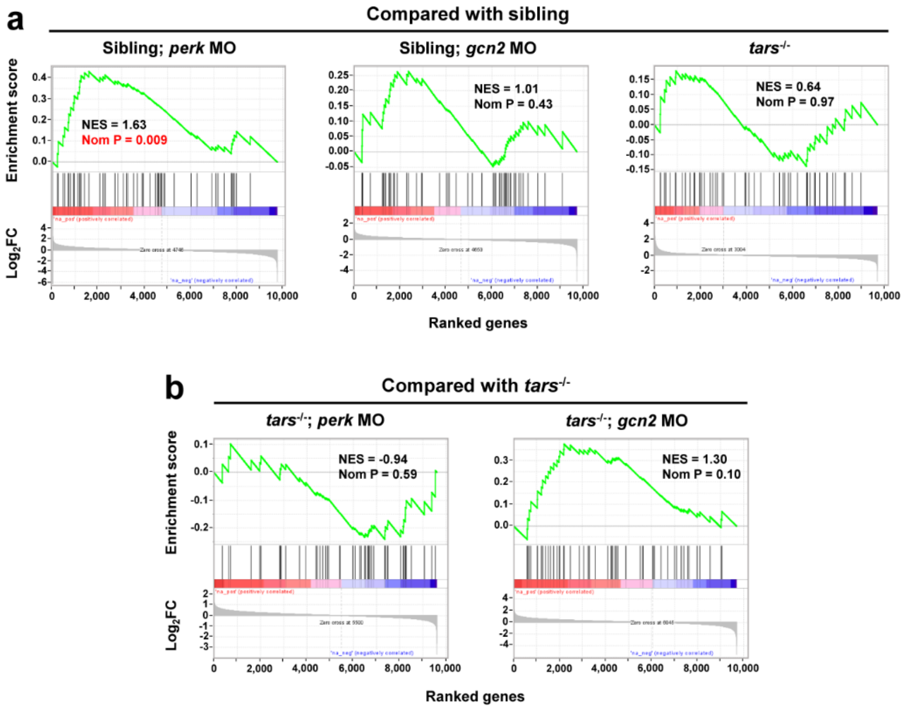

Supplementary Fig. S3 Perk functions predominantly in normal development but is overwhelmed in *tars*$^{-/-}$ stress condition. a Representative gene set enrichment analysis (GSEA) showing that knockdown of Perk in normal embryos (siblings) significantly activates RNA degradation-associated genes, as represented by the KEGG_RNA_DEGRADATION gene set (*left*). In contrast, knockdown of Gcn2 (*gcn2* MO) or knockout of Tars (*tars*$^{-/-}$) shows no significant effect (*middle* and *right*). The statistical significance of the enrichment in the GSEA is assessed by the Nominal p-value (Nom P) and the significant one (Nom P < 0.05) is written in *red*. **b** Knockdown of Perk (*left*) or Gcn2 (*right*) in the *tars*$^{-/-}$ embryos cannot activate the RNA degradation-associated genes.

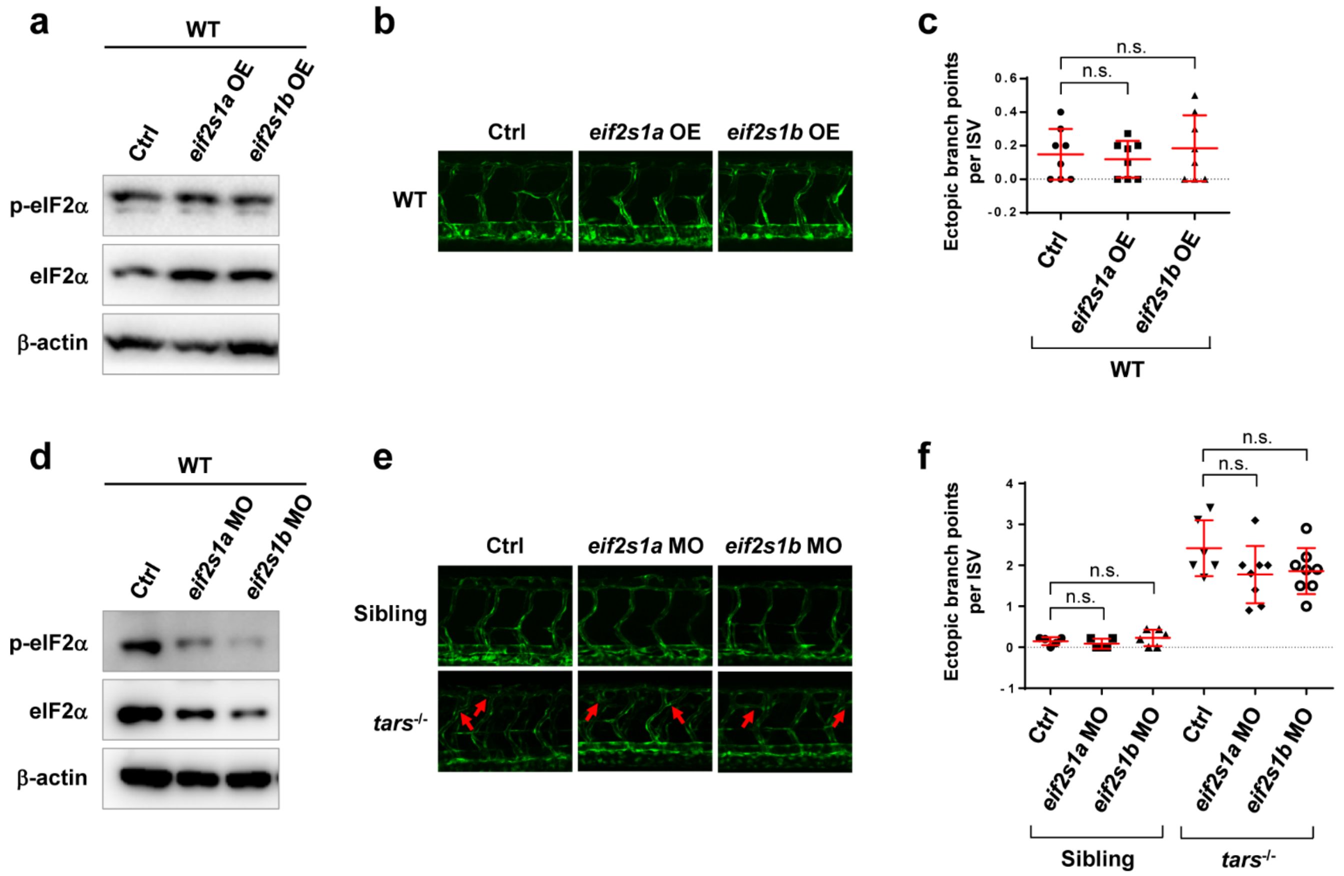

Supplementary Fig. S4 Changes in total eIF2α protein level show little effect on angiogenesis. a Immunoblot analysis of the eIF2α overexpressing (OE) WT embryos that were injected with *eif2s1a* or *eif2s1b* mRNAs. Note that, while the total eIF2α is increased, the phosphorylated eIF2α (p-eIF2α) is not significantly affected. **b** Confocal microscopy imaging of EGFP-labelled blood vessels in the trunk of the eIF2α OE embryos. **c** Quantification and statistical analysis of the ectopic branch points per intersomitic vessel (ISV) showing no significant difference between the control and eIF2α OE embryos. **d** Immunoblot analysis validating the efficiencies of the *eif2s1a* and *eif2s1b* morpholinos (MOs) in the WT embryos. **e**, **f** Confocal microscopy imaging and statistical analysis of the angiogenic phenotypes of the control and MO-injected sibling and *tars*$^{-/-}$ embryos. Note that the decrease of total eIF2α has no effect in the sibling embryos, and it very slightly, albeit not significantly, decreases the ectopic branch points in the *tars*$^{-/-}$ embryos.

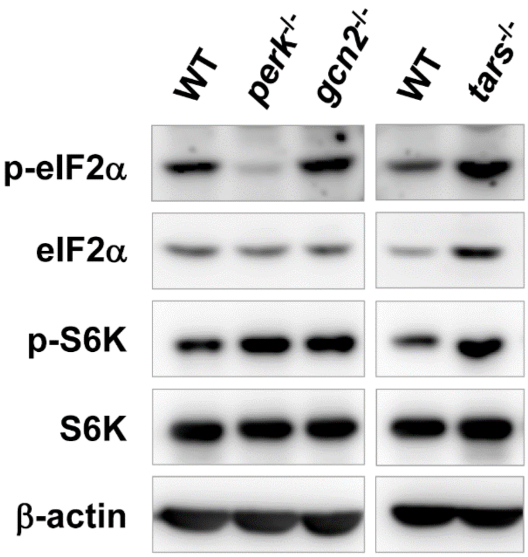

Supplementary Fig. S5 Immunoblot analysis of eIF2α phosphorylation in *perk*, *gcn2* and *tars* knockout zebrafish embryos. Note that the eIF2α phosphorylation (p-eIF2α) level is decreased in the *perk*$^{-/-}$, increased in the *tars*$^{-/-}$, and not changed in the *gcn2*$^{-/-}$ embryos. The embryos used in this experiment were all 3 dpf. Total eIF2α, phosphorylated S6K (p-S6K) and total S6K were also analyzed. β-actin was used as loading control.

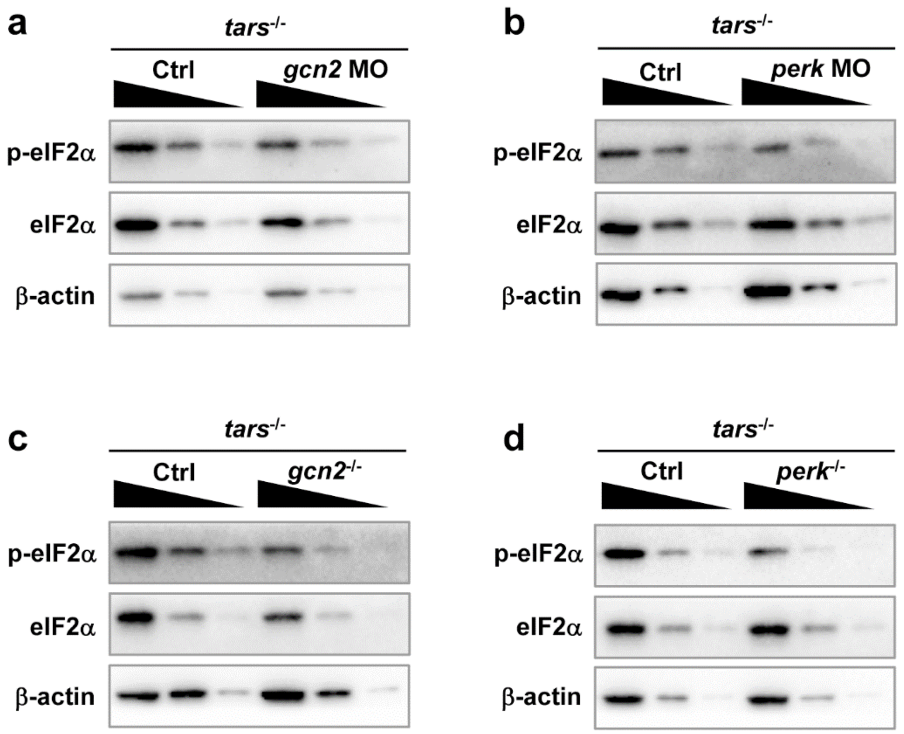

Supplementary Fig. S6 Serial dilution immunoblot analysis showing that Gcn2- and Perk-knockdown/knockout in the *tars*$^{-/-}$ embryos reduce eIF2α phosphorylation to a comparable extent. **a, b** The *tars*$^{-/-}$ embryos were injected with *gcn2* or *perk* MOs to knock down Gcn2 or Perk, respectively. **c, d** The *gcn2*$^{-/-}$ or *perk*$^{-/-}$ lines were crossed with *tars*$^{+/-}$ to obtain the double knockout embryos, which were compared with the *tars*$^{-/-}$ embryos. Each sample was loaded in 3-fold serial dilution to facilitate quantification, and β-actin was used as a loading control.

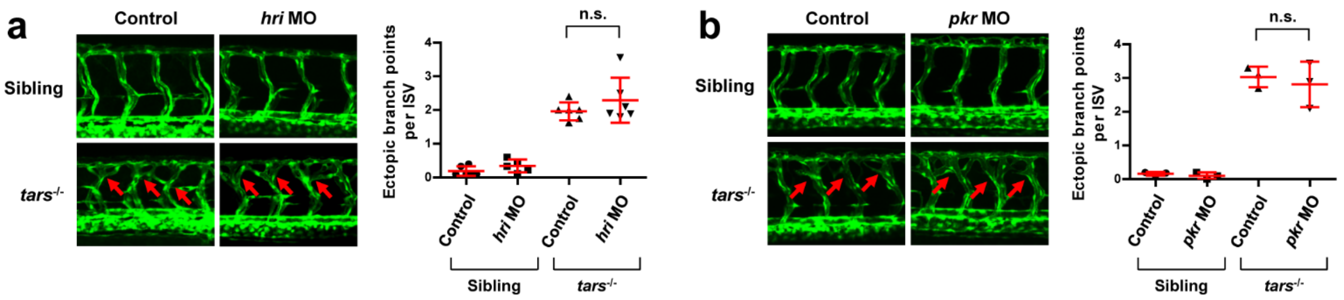

Supplementary Fig. S7 Knockdown of two other eIF2α kinases cannot rescue the angiogenic phenotypes in the *tars*$^{-/-}$ embryos. a, b Confocal microscopy imaging of EGFP-labelled blood vessels in the trunk of the *tars*$^{-/-}$ and sibling zebrafish embryos that were treated with morpholino that leads to knockdown of Hri (*hri* MO) or Pkr (*pkr* MO). Quantification and statistical analysis of the ectopic branch points per intersomitic vessel (ISV) of the embryos are shown in the *right*.

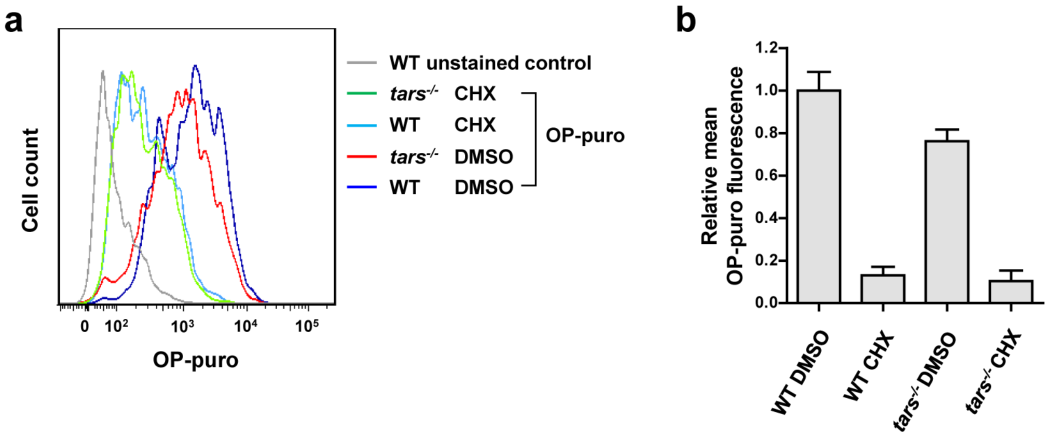

Supplementary Fig. S8 OP-puro incorporation assay results showing the slight decrease of global protein translation level in the *tars*$^{-/-}$ embryos. a Representative histogram plots of the OP-puro incorporation assay measuring the OP-puro fluorescence of the cells of WT and *tars*$^{-/-}$ embryos with and without cycloheximide (CHX) treatment. The embryos treated with CHX were used as a positive control because CHX was known to inhibit protein translation. The concentration of CHX was 360 μM. DMSO was used as solvent and was added into the control samples at the same final concentration. **b** Quantification of the relative mean OP-puro fluorescence values of the indicated samples. Data are presented as mean ± SD of three measurements.

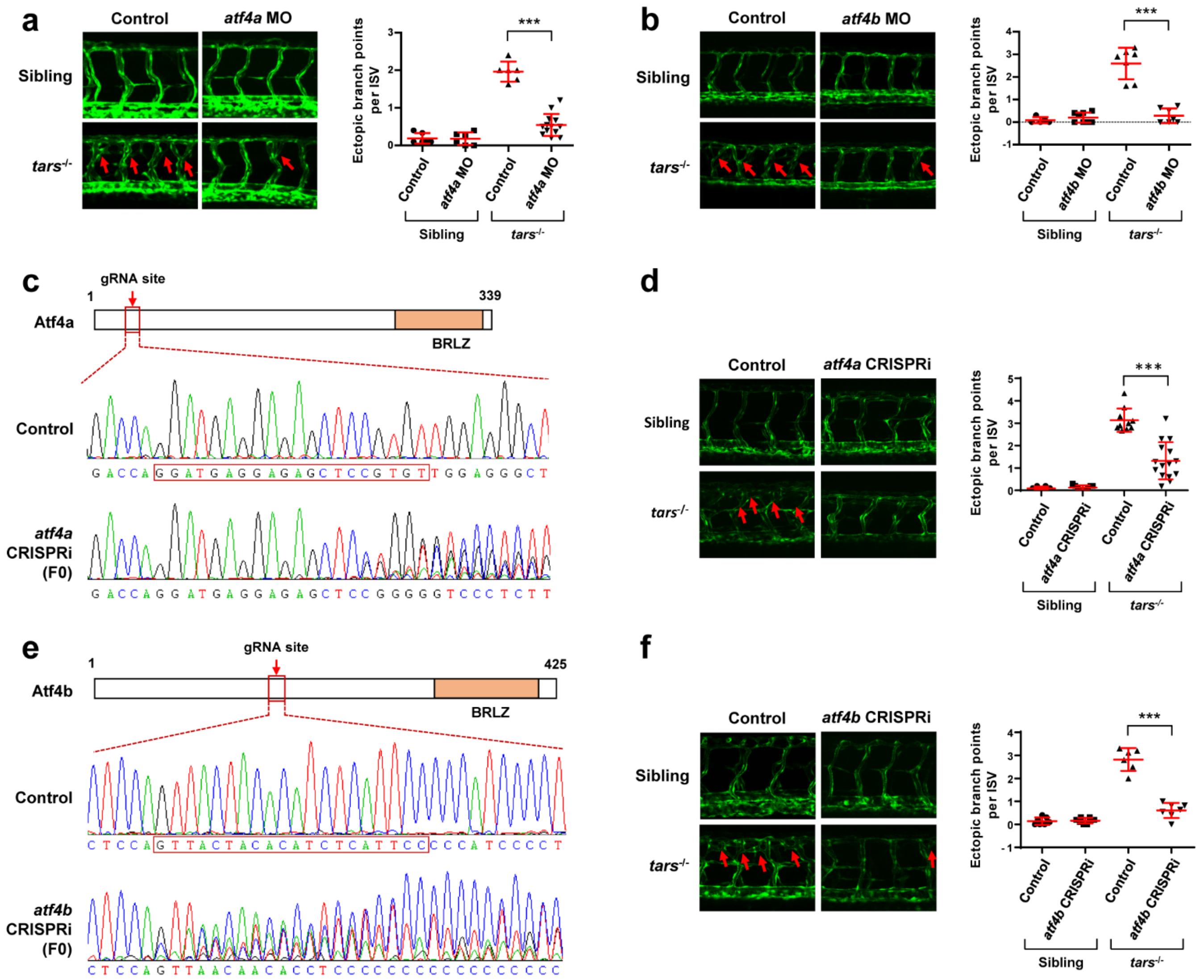

Supplementary Fig. S9 Role of Atf4 in the *tars*-deficiency-induced angiogenesis. a, b Morpholino-mediated knockdown of Atf4a (*atf4a* MO) and Atf4b (*atf4b* MO) significantly reduce the ectopic branch points of the blood vessels in the *tars*$^{-/-}$ embryos, indicative of a rescue of the angiogenic phenotypes. **c** CRISPR interference (CRISPRi) of Atf4a. The gRNA-targeting sequence (red box) is designed far upstream of the DNA-binding BRLZ (basic region leucin zipper) domain. The lower panel shows a sequencing result the F0 embryos, indicating efficient mutagenesis. **d** The CRISPRi of Atf4a significantly reduces the ectopic branch points of the blood vessels in the *tars*$^{-/-}$ embryos. **e, f** CRISPRi of Atf4b and its rescue of the angiogenic phenotypes. Data are presented as mean ± SD; ****P* < 0.001.

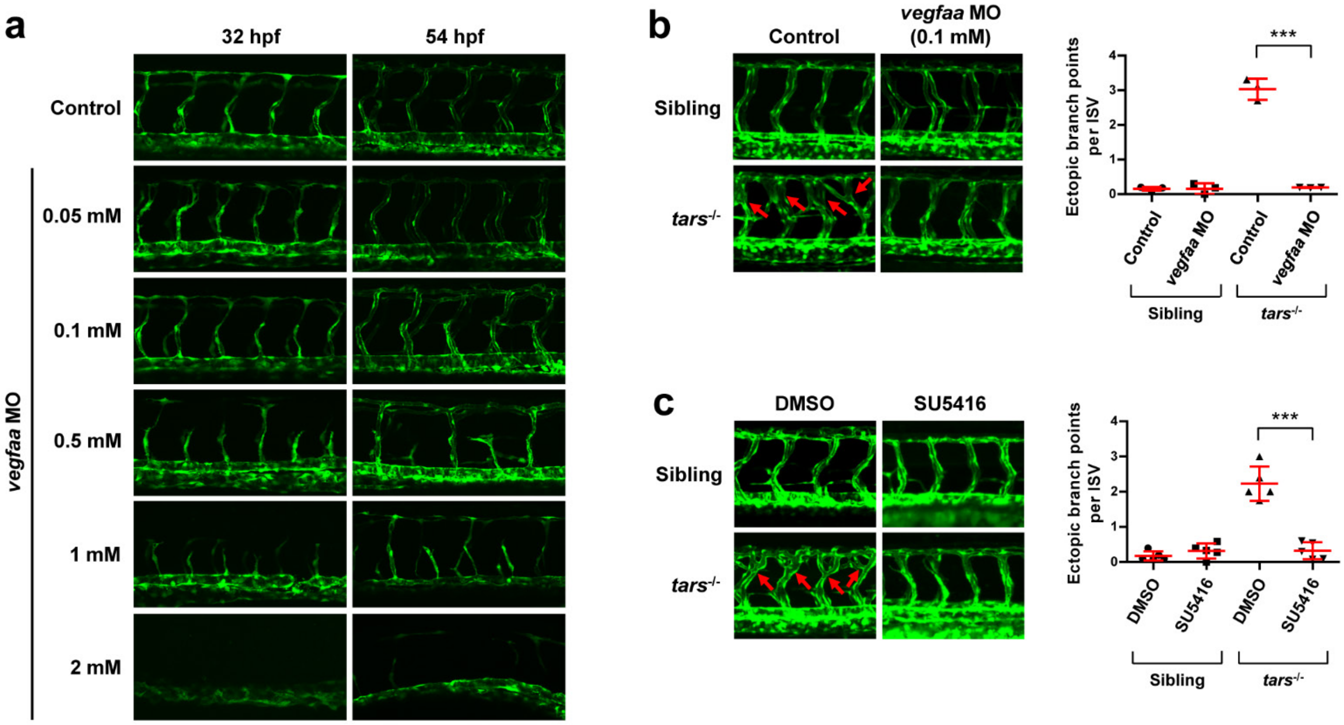

Supplementary Fig. S10 Role of Vegfα in the *tars*-deficiency-induced angiogenesis. a The *vegfaa* morpholino-injected embryos show a dosage-dependent inhibition of ISV growth, which phenocopies the previously reported *vegfaa* knockout line. **b** Injection of relatively lower dosage of *vegfaa* MO rescues the angiogenic phenotypes in the *tars*$^{-/-}$ embryos. **c** Pharmacological inhibition of Vegf receptor by SU5416 also significantly rescues the angiogenic phenotypes in the *tars*$^{-/-}$ embryos. Data are presented as mean ± SD; ****P* < 0.001.

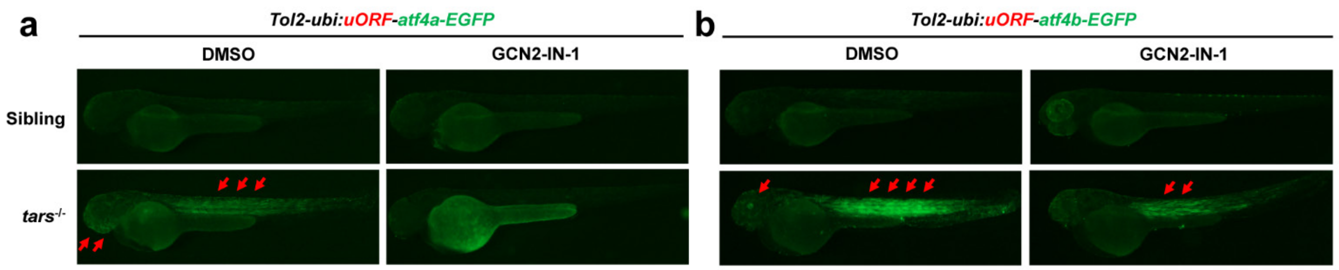

Supplementary Fig. S11 The Gcn2 inhibitor GCN2-IN-1 suppresses the enhanced translation of the *atf4a*- and *atf4b-EGFP* fusion ORFs in the *tars*$^{-/-}$ embryos. a, b Fluorescence microscope images showing that the enhanced translation levels of the *atf4a*- and *atf4b-EGFP* fusion ORFs in the truck and head of *tars*$^{-/-}$ embryos (red arrows) were inhibited by GCN2-IN-1. The Tol2 transposase-based transgenic system containing a *ubiquitin* promoter (*ubi:*) was used to drive the expression of the reporters.

Supplementary Tables

Supplementary Table S1 The KRIGE_AMINO_ACID_DEPRIVATION gene set, zebrafish ortholgogues and their expression levels (FPKM) in the embryos of indicated genotypes and treatments.

Human genes	Zebrafish orthologues	Sibling			*tars*$^{-/-}$		
		Control	*gcn2* MO	*perk* MO	Control	*gcn2* MO	*perk* MO
ASNS	*asns*	12.7778	13.499	17.4766	55.805	22.0833	74.541
ASS1	*ass1***						
ATF3	*atf3*	4.64294	2.67896	6.56419	26.8766	9.33584	18.6064
ATF5	*atf5a*	6.8079	5.2392	16.1516	27.5749	22.1082	41.73
	atf5b	10.8794	8.53554	34.0625	67.6599	52.4198	76.4682
CARS	*cars*	12.2941	11.1855	13.3785	27.5841	16.6269	29.5621
CBS	*cbsb*	23.6708	33.608	44.1811	96.7335	56.8833	143.436
	cbsa	0.992865	1.18739	2.64271	0.918626	1.49547	0.852058
CCNG2	*ccng2*	16.7789	12.6415	9.80828	17.0243	11.6678	17.1183
CDKN1A	*cdkn1a*	2.17782	8.88066	56.9732	6.20042	56.0636	13.6561
CEBPB	*cebpb*	36.2907	28.638	38.0368	81.1664	42.9882	66.5645
CHAC1	*chac1*	132.022	87.945	118.068	616.506	150.229	511.664
CLEC7A	*n.a.*						
CTH	*cth*	13.5546	15.6478	16.2564	45.2045	18.71	61.4893
	*cthl**	0.043517	0.077838	0.137926	0.087375	0.20336	0.130401
DDIT3	*ddit3*	14.2811	6.61826	29.4444	47.8308	42.9428	34.0392
DDIT4	*ddit4*	11.2316	5.46713	9.21389	21.0929	8.60009	11.1535
FYN	*fynb*	5.25218	3.44139	3.79559	2.96999	3.83948	3.84351
GADD45A	*gadd45ab*	5.90753	5.83784	9.31926	14.6411	12.8189	11.4121
	gadd45aa	2.92585	4.35368	9.83301	3.69133	9.80174	4.854
IL8	*n.a.*						
MARS	*mars*	12.8249	15.1477	20.9553	32.6454	24.3334	44.6962
PPP1R15A	*ppp1r15a*	15.3882	9.32604	9.59315	27.0431	11.2691	19.6778
PSAT1	*psat1*	45.2593	38.7016	42.9749	232.206	66.5781	291.449
RETN	*n.a.*						
SARS	*sars*	31.9545	31.0109	30.1541	57.1626	32.5118	59.4423
SESN2	*sesn2*	5.18575	3.13054	4.62645	48.8341	8.63054	44.6323
SLC38A2	*slc38a2*	23.8182	9.44088	9.79865	46.0872	14.8008	35.965
SLC7A11	*slc7a11**	0.192269	0.219077	0.102542	0.610412	0.263728	0.566294
STC2	*stc2a*	2.53331	2.59143	3.54231	33.7373	6.63798	28.1475
TRIB3	*trib3*	28.67	18.4501	20.2941	103.988	26.9422	86.237
VEGFA	*vegfaa*	4.40415	4.49817	5.32626	16.8601	7.45147	16.0333
	vegfab	3.77377	4.26537	3.86153	5.77992	4.62035	5.72762
WARS	*wars*	19.7912	22.5991	25.0422	42.9704	30.9267	50.2069

* Zebrafish gene(s) that was not included in the cluster analysis because its expression level was low (FPKM < 1) and thus presented a low signal-to-noice ratio.

** Zebrafish gene(s) that was absent from the analyzed version of ENSEMBL database.

n.a., not available; meaning that no zebrafish orthologue can be defined.

Supplementary Table S2 The REACTOME_PERK_REGULATED_GENE_EXPRESSION gene set, zebrafish ortholgogues and their expression levels (FPKM) in the embryos of indicated genotypes and treatments.

Human genes	Zebrafish orthologues	AAR-involved[a]	Reference (PMID)[a]	Sibling			*tars*$^{-/-}$		
				Control	*gcn2* MO	*perk* MO	Control	*gcn2* MO	*perk* MO
ASNS	*asns*	yes	Krige et al., 2008[1]	12.7778	13.499	17.4766	55.805	22.0833	74.541
ATF3	*atf3*	yes	Krige et al., 2008[1]	4.64294	2.67896	6.56419	26.8766	9.33584	18.6064
ATF4	*atf4a*	yes	Peng et al., 2002[2]	169.367	148.676	214.045	282.497	231.694	269.712
	atf4b	yes	Peng et al., 2002[2]	152.721	107.125	312.962	375.903	399.668	333.515
ATF4P3	*n.a.*								
ATF6	*atf6*			12.442	11.0678	10.9566	16.8622	13.3997	22.8186
CCL2	*n.a.*								
DCP2	*dcp2*			13.3283	12.2076	10.0888	10.8556	10.5474	11.1757
DDIT3	*ddit3*	yes	Krige et al., 2008[1]	14.2811	6.61826	29.4444	47.8308	42.9428	34.0392
DIS3	*dis3*			4.55522	5.60601	6.86919	3.83042	6.67433	4.77681
EIF2AK3	*eif2ak3*			10.2122	10.3506	8.52565	8.95028	8.99864	10.6469
EIF2S1	*eif2s1a*	yes	Peng et al., 2002[2]	33.0029	34.4637	38.5197	30.9618	33.8691	29.1565
	eif2s1b	yes	Peng et al., 2002[2]	113.559	121.382	125.093	187.983	132.115	192.38
EXOSC1	*exosc1*			14.8726	12.008	16.3129	13.3179	19.1027	13.8238
EXOSC2	*exosc2*			14.9154	16.227	26.1832	16.3332	24.628	15.958
EXOSC3	*exosc3*			11.5068	13.6581	14.4402	8.85049	11.8448	13.603
EXOSC4	*exosc4*			21.0085	21.9783	32.7858	18.6403	31.2613	15.2796
EXOSC5	*exosc5*			11.9159	14.6059	24.4431	12.2122	20.8276	11.7309
EXOSC6	*exosc6*			28.6422	25.8924	40.9818	22.7782	34.5389	14.8706
EXOSC7	*exosc7*			11.242	13.394	19.1051	11.9156	16.7994	10.7742
EXOSC8	*exosc8*			8.30142	12.069	16.4971	8.32502	14.577	9.51768
EXOSC9	*exosc9*			8.54771	7.94716	10.4738	7.77676	12.218	7.25746
HERPUD1	*herpud1*			7.1064	6.15103	5.8655	11.3432	6.48103	10.3902
HSPA5	*hspa5*			156.992	164.491	112.251	124.906	93.7419	114.531
IGFBP1	*igfbp1a*	yes	Jousse et al., 1998[3]	14.5027	9.03908	22.674	153.898	36.1958	159.483
	*igfbp1b**			1.01003	0.855965	0.516334	0.85408	0.280475	0.447482
IL8	*n.a.*								
KHSRP	*khsrp*			79.2965	85.0455	85.9661	67.376	72.4118	71.8212
LOC730136	*n.a.*								
NFYA	*nfyal*			4.22319	3.91737	4.03912	4.14596	4.0228	4.39618
	nfya			33.5126	29.3149	28.0291	33.3274	28.3857	33.9715
NFYB	*nfyba*			58.4525	58.6865	39.0586	52.0811	41.5343	52.4005
	nfybb			5.4887	5.70052	5.54155	5.03619	5.53048	7.58983
PARN	*parn*			25.4634	23.5101	22.6184	24.2401	21.7613	20.856

[a] Some genes of this geneset is also involved in the AAR pathway, as described in the indicated reference.

* Zebrafish gene(s) that was not included in the cluster analysis because its expression level was low (FPKM < 1) and thus presented a low signal-to-noice ratio.

n.a., not available; meaning that no zebrafish orthologue can be defined.

Supplementary Table S3 The GO_IRE1_MEDIATED_UNFOLDED_PROTEIN_RESPONSE gene set, zebrafish ortholgogues and their expression levels (FPKM) in the embryos of indicated genotypes and treatments.

Human genes	Zebrafish orthologues	Sibling			*tars*$^{-/-}$		
		Control	*gcn2* MO	*perk* MO	Control	*gcn2* MO	*perk* MO
ACADVL	*acadvl*	27.4021	30.6963	22.1702	25.6829	23.5006	28.9751
ADD1	*add1*	21.1272	18.3327	16.0684	13.9502	18	16.1944
ARFGAP1	*CABZ01091853.1***						
ASNA1	*asna1*	14.7906	14.8294	16.4709	12.7717	15.6203	12.5889
ATP6V0D1	*atp6v0d1*	27.1458	23.7058	22.6956	23.3905	22.5548	22.8995
C19orf10	*mydgf*	19.4295	22.3921	19.7122	14.8827	18.4382	17.9356
CTDSP2	*ctdsp2*	26.3171	31.2973	29.1188	33.4074	33.4835	44.1033
CUL7	*n.a.*						
CXXC1	*cxxc1a*	6.86687	7.00419	5.65863	6.31773	5.39455	6.27812
	cxxc1b	9.16509	8.8912	7.36467	9.51463	7.43676	7.05097
DCTN1	*dctn1b*	2.11174	1.64734	1.19003	1.83618	1.35405	1.61869
DDX11	*ddx11*	2.4044	2.66523	3.56883	2.02473	2.69864	2.75115
DNAJB11	*dnajb11*	20.13	19.5681	14.2497	14.8184	12.6073	13.2893
DNAJB9	*dnajb9a*	4.5605	2.44719	1.87907	2.60458	2.37793	2.61167
DNAJC3	*dnajc3a*	5.77002	6.40965	4.00289	5.17343	2.82932	4.35697
EDEM1	*edem1*	8.88653	8.15055	7.61754	7.05475	7.5346	6.59871
ERN1	*ern2*	1.01751	1.46251	1.66768	0.668838	1.50827	1.27042
EXTL3	*extl3*	6.1502	5.13564	5.17431	3.67	5.84246	4.24995
FKBP14	*fkbp14*	11.8696	14.4703	14.0084	10.3045	14.7785	14.0823
GFPT1	*gfpt1*	14.1673	15.4687	16.374	11.3158	16.562	15.5291
GOSR2	*gosr2*	16.0088	19.9361	15.1976	17.9523	14.0263	16.4757
GSK3A	*gsk3ab*	34.0197	29.4933	30.5376	32.1645	31.2777	28.2231
	gsk3aa	5.7861	4.86854	4.43027	4.82346	5.73406	4.41911
HDGF	*n.a.*						
HSPA5	*hspa5*	156.992	164.491	112.251	124.906	93.7419	114.531
HYOU1	*hyou1*	19.1991	21.0289	17.2616	18.5432	15.5348	22.0831
KDELR3	*kdelr3*	17.8931	25.0381	15.7992	15.2845	15.2683	19.9886
KLHDC3	*klhdc3*	21.4452	23.9742	25.1428	24.9407	23.975	22.6426
LMNA	*lmna*	4.78528	4.88764	6.62941	4.17122	6.36784	5.75813
PDIA5	*pdia5*	13.3178	17.014	16.4621	11.3725	17.5973	14.5372
PDIA6	*pdia6*	39.7802	50.748	31.9132	25.4756	26.9681	33.4494
PLA2G4B	*n.a.*						
PPP2R5B	*PPP2R5B*	4.57614	3.0562	2.54514	4.18589	2.11948	2.39568
PREB	*preb*	10.0795	12.0888	13.945	11.5267	13.3328	11.3745
PTPN1	*ptpn1*	17.7697	14.3358	18.5679	17.4036	19.4374	15.3843
SEC31A	*sec31a*	7.93099	9.80757	8.07053	6.4381	7.96626	8.91037
SEC61A1	*sec61a1l*	16.3911	18.6649	15.1938	14.6405	14.2771	14.2597
	sec61a1	81.7947	86.3473	71.2337	77.6719	66.6339	82.545
SEC61A2	*n.a.*						
SEC61B	*sec61b*	66.2475	70.2424	60.0764	69.2136	57.2728	66.8648
SEC61G	*sec61g*	115.504	139.127	111.67	124.96	104.519	128.034

SEC62	*sec62*	21.0845	17.6026	18.3897	18.9085	19.6056	13.8231
SEC63	*sec63*	14.6637	13.2114	11.6047	11.8096	12.753	11.8492
SERP1	*serp1*	88.7814	94.0731	91.2115	89.5904	89.5481	93.4526
SHC1	*shc1*	15.7794	14.4935	12.9485	13.2657	13.4146	14.2615
SRPR	*srpr*	21.8308	19.0865	18.5546	21.79	18.0502	23.8366
SRPRB	*srprb*	16.617	20.1562	28.3389	18.1783	23.4701	19.6338
SSR1	*ssr1*	112.396	139.154	125.824	115.889	115.749	120.108
SULT1A3	*n.a.*						
SYVN1	*syvn1*	13.809	12.4166	11.8996	13.1871	9.72484	11.3123
TATDN2	*tatdn2*	0.186181	0.145696	0.159818	0.062303	0.114479	0.05424
TLN1	*tln1*	14.2165	14.3997	14.9725	12.5663	16.0774	14.8673
TPP1	*tpp1*	12.7414	12.2376	13.4584	11.4731	13.1779	12.0034
TSPYL2	*n.a.*						
WFS1	*wfs1b*	1.51104	1.07594	1.754	1.39027	1.36112	1.33786
WIPI1	*wipi1*	12.58	15.1905	10.4325	20.3661	13.6742	29.4787
XBP1	*xbp1*	123.694	126.274	114.142	244.094	127.903	253.031
YIF1A	*yif1a*	11.6937	15.5337	16.1823	11.3265	16.5869	13.5465
ZBTB17	*zbtb17*	11.2168	5.6836	9.70476	10.3418	11.5698	6.66083

** Zebrafish gene(s) that was absent from the analyzed version of ENSEMBL database.

n.a., not available; meaning that no zebrafish orthologue can be defined.

Supplementary Table S4 The REACTOME_ACTIVATION_OF_CHAPERONES_BY_ATF6_ALPHA gene set, zebrafish ortholgogues and their expression levels (FPKM) in the embryos of indicated genotypes and treatments.

Human genes	Zebrafish orthologues	AAR-involved[a]	Reference (PMID)[a]	Sibling			*tars*$^{-/-}$		
				Control	*gcn2* MO	*perk* MO	Control	*gcn*2 MO	*perk* MO
ATF4	*atf4a*	yes	Peng et al., 2002[2]	169.37	148.68	214.05	282.5	231.69	269.71
	atf4b	yes	Peng et al., 2002[2]	152.72	107.13	312.96	375.9	399.67	333.52
ATF4P3	*n.a.*								
ATF6	*atf6*			12.442	11.068	10.957	16.862	13.4	22.819
CALR	*calr*			36.552	43.123	21.445	26.845	18.807	30.425
DDIT3	*ddit3*	yes	Krige et al., 2008[1]	14.281	6.6183	29.444	47.831	42.943	34.039
HSP90B1	*hsp90b1*			60.349	76.837	38.669	42.559	33.541	45.17
HSPA5	*hspa5*			156.99	164.49	112.25	124.91	93.742	114.53
LOC730136	*n.a.*								
MBTPS1	*mbtps1*			10.578	11.785	10.573	9.9886	10.945	11.714
MBTPS2	*mbtps2*			4.9965	5.2869	5.3737	4.5296	4.8088	5.2161
NFYA	*nfyal*			4.2232	3.9174	4.0391	4.146	4.0228	4.3962
	nfya			33.513	29.315	28.029	33.327	28.386	33.972
NFYB	*nfyba*			58.453	58.687	39.059	52.081	41.534	52.401
	nfybb			5.4887	5.7005	5.5416	5.0362	5.5305	7.5898
XBP1	*xbp1*			123.69	126.27	114.14	244.09	127.9	253.03

[a] Some genes of this geneset is also involved in the AAR pathway, as described in the indicated reference.

n.a., not available; meaning that no zebrafish orthologue can be defined.

Supplementary Table S5 Sequences of the oligonucleotides used in this study.

oligonucleotide	Sequence	Source
**Primers for genotyping**		
tars-genotyping-for	ATTTGAAGCTGACAGGGA	
tars-genotyping-rev	ACCGAAGTAATGAGAAGGAT	
gcn2-genotyping-for	ACTGTGGTGCACAAGCAAAG	
gcn2-genotyping-rev	CCGACTCACTCCTCCAAAAC	
perk-genotyping-for	AACACTGCTTTATTTGCACATCT	
perk-genotyping-rev	TAAGGAAATGGGTGGTCCTG	
**Primers for RT-qPCR**		
Zebrafish-*atf3*-for	CTGTCCCAGAGGAGAACGAC	
Zebrafish-*atf3*-rev	GCTCTGCATTGATGGACTCA	
Zebrafish-*atf4a*-for	CTTTCTCTCCTCCTGCTTCT	
Zebrafish-*atf4a*-rev	GAGTCACACGACCCAATCA	
Zebrafish-*vegfaa*-for	AAAAGAGTGCGTGCAAGACC	
Zebrafish-*vegfaa*-rev	GACGTTTCGTGTCTCTGTCG	
Zebrafish-*asns*-for	TGCCTTCTCTCAGGTGGTCT	
Zebrafish-*asns*-rev	CATCTGGACTGTCCTCAGCA	
Zebrafish-*cars*-for	TCAGTGCTGTCCGATTTCAG	
Zebrafish-*cars* -rev	ACCCCCAGCTCAGGTAAAGT	
Zebrafish-*sars*-for	GTGGCTGAAGCCAGAAGAAC	
Zebrafish-*sars*-rev	GGCGTACACAAACTGCTCAA	
Zebrafish-*mars*-for	GCTGAAGTGCATCCTCAACA	
Zebrafish-*mars*-rev	GCCACATTCACTGACACACC	
Zebrafish-*β-actin*-for	TGCTGTTTTCCCCTCCATTG	
Zebrafish-*β-actin*-rev	TTCTGTCCCATGCCAACCA	
**Morpholino oligonucleotides (MOs)**		
tars MO	GATCAGTCACACTCTCATCCGCCAT	Castranova et al., 2016[4]
gcn2 MO	TCATCCTTCATTCATCTTTCTTCGT	This paper
perk MO	ACTGAAACCCCCTTTCCATTGGGAC	Jia et al., 2015[5]
hri MO	CTCCGTATCTGTCAGGCTGAACATT	This paper
pkr MO	TTTCCTGACAGAGACTCCATTGCGA	This paper
eif2s1a MO	CTGGCATCTTCACCCGATATGTAGG	This paper
eif2s1b MO	AAAACCGACAGCTCAGACCCGGCAT	This paper
atf4a MO	CAGCGTCCCCAACACACAGAGACAT	Castranova et al., 2016[4]
atf4b MO	CATGGCTGTCTATTTCAAGTGGAAT	This paper
vegfaa MO	TAAGAAAGCGAAGCTGCTGGGTATG	Nasevicius et al., 2000[6]
**DIG-labeled probes for Northern Blot**		
Zebrafish-tRNA^{Thr}(UGU)	CCCAGCGAGGATCGAACTCGCGCCCCTG	This paper
Zebrafish-tRNA^{Thr} (AGU/CGU)	CTTTACCAACTAAGCCACA	This paper
Zebrafish-tRNA^{Gly}GCC	CGCGTGGCAGGCGAGAATTC	This paper
Zebrafish-5S rRNA	GCAACCTAGTTTTCCCATGTGGTCTCCAT	This paper

Supplementary References

1 Krige, D. *et al.* CHR-2797: an antiproliferative aminopeptidase inhibitor that leads to amino acid deprivation in human leukemic cells. *Cancer Res* **68**, 6669-6679 (2008).

2 Peng, T., Golub, T. R. & Sabatini, D. M. The immunosuppressant rapamycin mimics a starvation-like signal distinct from amino acid and glucose deprivation. *Mol Cell Biol* **22**, 5575-5584 (2002).

3 Jousse, C., Bruhat, A., Ferrara, M. & Fafournoux, P. Physiological concentration of amino acids regulates insulin-like-growth-factor-binding protein 1 expression. *Biochem J* **334**, 147-153 (1998).

4 Castranova, D. *et al.* Aminoacyl-Transfer RNA Synthetase Deficiency Promotes Angiogenesis via the Unfolded Protein Response Pathway. *Arterioscler Thromb Vasc Biol* **36**, 655-662 (2016).

5 Jia, X. E. *et al.* Mutation of kri1l causes definitive hematopoiesis failure via PERK-dependent excessive autophagy induction. *Cell Res* **25**, 946-962 (2015).

6 Nasevicius, A., Larson, J. & Ekker, S. C. Distinct requirements for zebrafish angiogenesis revealed by a VEGF-A morphant. *Yeast* **17**, 294-301 (2000).
